# Oxidative stress-induced mutagenesis in single-strand DNA occurs primarily at cytosines and is DNA polymerase zeta-dependent only for adenines and guanines

**DOI:** 10.1093/nar/gkt671

**Published:** 2013-08-07

**Authors:** Natalya P. Degtyareva, Lanier Heyburn, Joan Sterling, Michael A. Resnick, Dmitry A. Gordenin, Paul W. Doetsch

**Affiliations:** ^1^Department of Biochemistry, ^2^Winship Cancer Institute, Emory University School of Medicine, 4013 Rollins Research Center, Atlanta, GA 30322, USA, ^3^Laboratory of Molecular Genetics, National Institute of Environmental Health Sciences (NIH, DHHS), Research Triangle Park, NC 27709, USA and ^4^Department of Radiation Oncology, Emory University School of Medicine, 4013 Rollins Research Center, Atlanta, GA 30322, USA

## Abstract

Localized hyper-mutability caused by accumulation of lesions in persistent single-stranded (ss) DNA has been recently found in several types of cancers. An increase in endogenous levels of reactive oxygen species (ROS) is considered to be one of the hallmarks of cancers. Employing a yeast model system, we addressed the role of oxidative stress as a potential source of hyper-mutability in ssDNA by modulation of the endogenous ROS levels and by exposing cells to oxidative DNA-damaging agents. We report here that under oxidative stress conditions the majority of base substitution mutations in ssDNA are caused by erroneous, DNA polymerase (Pol) zeta-independent bypass of cytosines, resulting in C to T transitions. For all other DNA bases Pol zeta is essential for ROS-induced mutagenesis. The density of ROS-induced mutations in ssDNA is lower, compared to that caused by UV and MMS, which suggests that ssDNA could be actively protected from oxidative damage. These findings have important implications for understanding mechanisms of oxidative mutagenesis, and could be applied to development of anticancer therapies and cancer prevention.

## INTRODUCTION

Localized somatic hyper-mutability is a phenomenon of accumulating multiple mutations in a small section of the genome. It may lead to a rapid increase in fitness (1) or contribute to tumorigenesis (2,3). Several lines of evidence suggest that the persistence of long stretches of single-stranded DNA (ssDNA) either in the vicinity of a double-strand break (DSB) or at uncoupled replication forks can lead to accumulation of such clustered mutations (2–6). Both acute and chronic exposure to exogenous or endogenous mutagenic factors increase the incidence of mutations and amplify the probability of clustered, multiple genomic variations allowing for survival and further clonal expansion of malignant cells (7). The inability of the majority of DNA repair systems to process lesions in single-stranded substrates is likely to increase the contribution of ssDNA damage to hyper-mutability. Therefore mechanisms and origins of spontaneous and induced mutagenesis ssDNA are of special interest.

The levels of spontaneous mutations in ssDNA can be orders of magnitude higher than in dsDNA (4,6). Hyper-mutability may be caused by increased rate of errors during re-synthesis of the second DNA strand and/or by lesions inflicted by the products of cellular metabolism. Exposure to DNA-damaging agents (MMS, UV-irradiation and sulfites) further amplifies hyper-mutability of ssDNA (6,8,9). Oxidative stress defines the shape of multiple biological processes since normal aerobic metabolism generates a highly reactive, oxidizing environment for all macromolecules in living organisms. Increased levels of reactive oxygen species (ROS) are implicated in the etiology and progression of pathological conditions such as cancer (10), chronic inflammation (11) and neurodegenerative diseases (12). Oxidative stress is known to be a threat to genomic integrity (13–15).

ROS damage DNA and produce dozens of chemically distinct lesions that distort the structure and coding properties of DNA and may cause mutations and chromosomal aberrations. Despite numerous reports on the correlation between elevated cellular levels of ROS and increased mutations and chromosome aberrations rates (14,16), the precise mechanisms of ROS-induced genome destabilization remain poorly understood. Delineation of the pathways, contributing to ROS-induced genome instability is complicated by several factors including (i) redundancy and overlapping specificities of DNA damage repair and tolerance pathways (17); (ii) the ability of the replication fork to bypass oxidative damage with relatively high efficiency (18,19); and (iii) the absence of simple and reliable methods for detection and quantification of oxidative DNA damage *in vivo* as well as the low sensitivity of existing methods for determining endogenous ROS levels.

The major confounding factor in defining the potential to generate mutagenic lesions and the mutagenic signature of both endogenous and exogenous oxidative damage appears to be the redundancy of pathways for repairing oxidative DNA damage. The majority of enzymes involved in the DNA base excision repair (BER) pathway, which is the major pathway for repair of oxidative DNA damage, have a broad spectrum of substrates and can replace each other at the initial steps of repair. Furthermore, when the capacity of BER is severely compromised, the nucleotide excision repair (NER) pathway, normally involved in processing of bulky DNA lesions can function in the repair of a sub-set of oxidative DNA lesions (17). To make the picture even more complex, the post-replicative mismatch repair system can also recognize and remove 8-oxoG-A mis-pairs (20). Therefore, defining an unaltered mutagenic signature of oxidative damage requires simultaneous elimination of multiple systems of protection acquired during the evolution of aerobic organisms. In principle, this can be achieved through analysis of the mutagenesis in strains in which multiple DNA damage-handling genes are compromised. Although such approaches has been used by several groups (21,22) it is likely, that misinterpretation of the mutation signature data occurs due to the indirect effects of multiple mutations in the cellular background. For example, a complete elimination of both BER and NER pathways in yeast requires disruption of at least four DNA repair genes. Such quadruple mutants show significant changes in transcription patterns of different genes (23,24), as compared to defects in either BER or NER caused by a single gene disruption. Such pleiotropic deregulation could significantly affect the mutation spectra, making it difficult to recapitulate the spectrum of oxidative mutagenic lesions in cells corrupted for both BER and NER. An alternative to inactivation of BER and NER would to utilize an ssDNA substrate that cannot be processed by the major DNA repair pathways even if those pathways are completely functional in the rest of the genome.

In this study we employed budding yeast strains with an ssDNA mutation reporter, which is not influenced by indirect effects and is free from ambiguity in determining the damaged nucleotide giving rise to a base substitution. This allowed generation of a more accurate picture of the mutagenic properties of ROS. We also assessed the relative contributions of oxidative stress to hyper-mutability and gross chromosomal rearrangements (GCRs) by measuring mutation frequencies and defining the signature of oxidative DNA damage in a sensitive ssDNA reporter system. Utilization of this tool allowed identification of mutagenesis-prone targets of oxidative damage and made it possible to compare the mutagenic impact of ROS on ssDNA to that of other types of DNA-damaging agents such as UV-irradiation, methyl methanesulfonate (MMS) and sulfites. We also addressed the role of translesion synthesis (TLS) polymerase zeta in oxidative stress-induced mutagenesis. Our results challenge several established views on mutagenesis in higher organisms and could have important implications for development of the therapeutic strategies aiming at selective killing of the cancer cells via increase in their mutational load.

## MATERIALS AND METHODS

### Genetic analysis and media

Genetic procedures (transformation, tetrad analysis, etc.), cell growth and selection conditions were performed as previously described (25). Strains were grown at 30°C. *cdc13-1ts* mutants were grown at room temperature (23°C). In experiments which required telomere-uncapping cells were incubated at 37°C.

### Strain construction

Yeast strains used in this study are isogenic to CG379 (26). The common genotype of haploid strains was *MATα his7-2 leu2-3,112 trp1-289 can1Δ cdc13-1 ura3Δ*. Strains also contained a *CAN1-URA3* mutation reporter similar to the one used by Chan *et al.* (9) but missing an insert of the *ADE2* open reading frame. *CAN1-URA3* reporter was placed either in sub-telomeric or in mid-chromosome position as described in Results section. To avoid accumulation of mutations during propagation of strains defective in DNA repair and/or ROS scavenging, we constructed a series of homozygous diploid strains heterozygous for mutations affecting ROS accumulation and/or DNA repair. These strains were built by first initiating mating type switching after introducing YEp-HO plasmid and then by series of consecutive gene disruptions in the resulting diploid strains JFS1327 and JFC1328, containing *CAN1-URA3* sub-telomeric reporter construct used to assess mutagenesis in ssDNA and in control diploid strains JFS1325 and JFS1326 with mid-chromosome location of *CAN1-URA3* reporter. The genes of interest were disrupted by transformation with the PCR fragments (primers sequences are available upon request) containing genes for antibiotic resistance flanked by the upstream and downstream sequences of the corresponding genes (Supplementary Table S5). Deletions were confirmed by PCR.

### Measurement of CAN1 mutation frequencies and frequencies of GCRs

Genetic defects leading to accumulation to ROS often lead to reduced growth rate increasing the probability of selecting a suppressor after long propagation. In order to reduce propagation before measuring mutation rates, diploids heterozygous for the gene of interest (Supplementary Table S5) were sporulated and dissected. Fresh spore colonies of the genotypes of interest were inoculated and grown for 72 h in rich liquid media. Following 1:10 dilution into rich media cells were incubated in a shaker at 37°C and/or 23°C for 3.5 h. Then cells were quickly washed with water, and exposed to oxidative-damaging agents (5 mM hydrogen peroxide or 150 µM paraquat in water) for 1 h at room temperature. Cells were washed in water and plated on synthetic complete medium containing canavanine and lacking arginine to measure the frequency of canavanine-resistant mutants. For *sod1Δ* cells canavanine-containing media was supplemented with L-methionine and L-lysine to final concentration of 200 mg/l and 300 mg/l, respectively. A diluted suspension was also plated on medium lacking canavanine to estimate number of cells in culture. At least four independent haploid spore isolates were examined for each genotype at any given experiment. The mutation frequencies were calculated as a ratio of cells resistant to canavanine to the total number of viable cells in culture. To estimate the frequencies of GCRs plates with Can^R^ clones were replica plated onto media lacking uracil. Can^R^ Ura^−^ clones from independent cultures were streaked on YPD and single colony was used to inoculate the culture from which genomic DNA was prepared using Epicentre MasterPure Yeast DNA Purification Kit (Illumina, Madison, WI). The loss of left arm of chromosome V was confirmed by PCR with the primers complimentary to telomere-proximal *URA3* sequence (oDG_97 5′-TGAAGAAGCTGCATTTGC-3′ and oDG_98 5′-TTGTTAGCGGTTTGAAGCAG-3′) and by CHEF as described in (16).

### Statistical methods

Statistical evaluations were done with non-parametric Fisher exact, chi-square and Mann–Whitney tests.

### Identification of the mutations in ssDNA reporter

Independent Can^R^ Ura^−^ or Can^R^ clones from cultures originated from several haploid isolates were streaked on YPD plates. After confirmation of the phenotype by replica plating on corresponding media, single colonies of interest were used to set up the cultures for purification of the genomic DNA as described above. *CAN1* or *CAN1* and *URA3* loci were PCR amplified and sequenced with primers listed in Supplementary Table S6. Complete list of mutations is presented in Supplementary Table S7. TaKaRa Ex Taq DNA polymerase (Takara Bio Inc., Japan) was used for PCR; manufacturer protocol was followed in choosing of the settings for the PCR reactions.

## RESULTS

### ROS causes mutations in ssDNA

**Figure 1.**
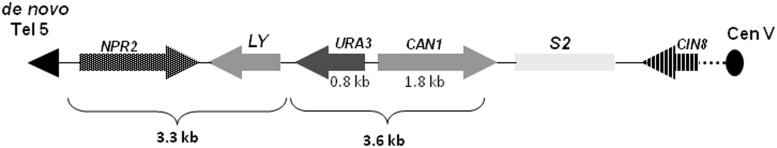
Schematic depiction of the reporter for ssDNA mutagenesis. *CAN1* and *URA3* open reading frames had been placed in the *LYS2* locus on chromosome V. Arrows indicate the direction of transcription of the genes. Left arm of *de novo* constructed telomere is represented by a triangle; an oval depicts the centromere.

Previous studies have indicated that the frequency of spontaneous mutagenesis in ssDNA can be two orders of magnitude higher, than in dsDNA (4,6). At least 90% of spontaneous mutations in ssDNA relied on error-prone TLS by DNA polymerase (Pol) zeta, which is essential for different kinds of damage-induced mutagenesis (reviewed in (6,27,28)). This suggests that endogenous DNA damage could be a predominant cause of spontaneous mutations in those experiments. We hypothesized that major contributors to spontaneous ssDNA hyper-mutability are ROS. To address this hypothesis we used a reporter system similar to those developed by Chan *et al.* (9), to measure mutation frequencies in ssDNA and to detect clusters of simultaneous mutations as changes in phenotypes controlled by two closely spaced ORFs. In these strains the wild-type *CAN1* and *URA3* genes on the left arm of chromosome V were deleted, the *CAN1* and *URA3* open reading frames were inserted into the *LYS2* gene placed within ∼8.9 kb from an artificially constructed telomere at the left tip of chromosome V (Figure 1). The DNA between the telomere and the *lys2::URA3-CAN1* in this construct does not contain any essential genes. The yeast strains carried a *cdc13-1* temperature sensitive mutation, conferring a defect in telomere capping. At the non-permissive temperature 37°C, 5′→3′ resection occurs, generating long stretches of ssDNA adjacent to telomeric regions (29,30); after 3.5 h cells arrest at G2. At that time cells were exposed to oxidative DNA-damaging agents for 1 h at room temperature. They were subsequently plated onto canavanine-containing media for mutant selection and, after dilution, onto rich growth media to determine cell viability. Mutation frequencies were calculated as a ratio of cells resistant to canavanine to the total number of viable cells in culture.

Since cells were not dividing when ssDNA was formed and throughout the time of exposure to endogenous and exogenous-damaging agents and restoration to dsDNA, these frequencies directly reflect the probability of mutant allele formation. Cells with an additional mutation in the *URA3* reporter gene were identified by replica plating of canavanine-resistant colonies onto selective media lacking uracil. As a control, the *URA3-CAN1* reporter was inserted into the *LYS2* gene located within its unperturbed linkage group in the middle of the right arm of chromosome II (2) to address mutation in dsDNA. The 345 kbp segment of DNA between this mid-chromosome reporter and telomere contains 35 essential genes, which eliminates the opportunity for survival of GCR events in this region. The long distance between the telomere and the reporter construct precludes a stretch of ssDNA from encompassing reporter genes after telomere uncappping at 37°C. In order to reduce the chance of selecting growth enhancers in slow growing mutants with elevated levels of ROS we measured mutation rates in at least six independent meiotic haploid isolates after sporulating heterozygous diploids (see ‘Materials and Methods’ section).

**Figure 2.**
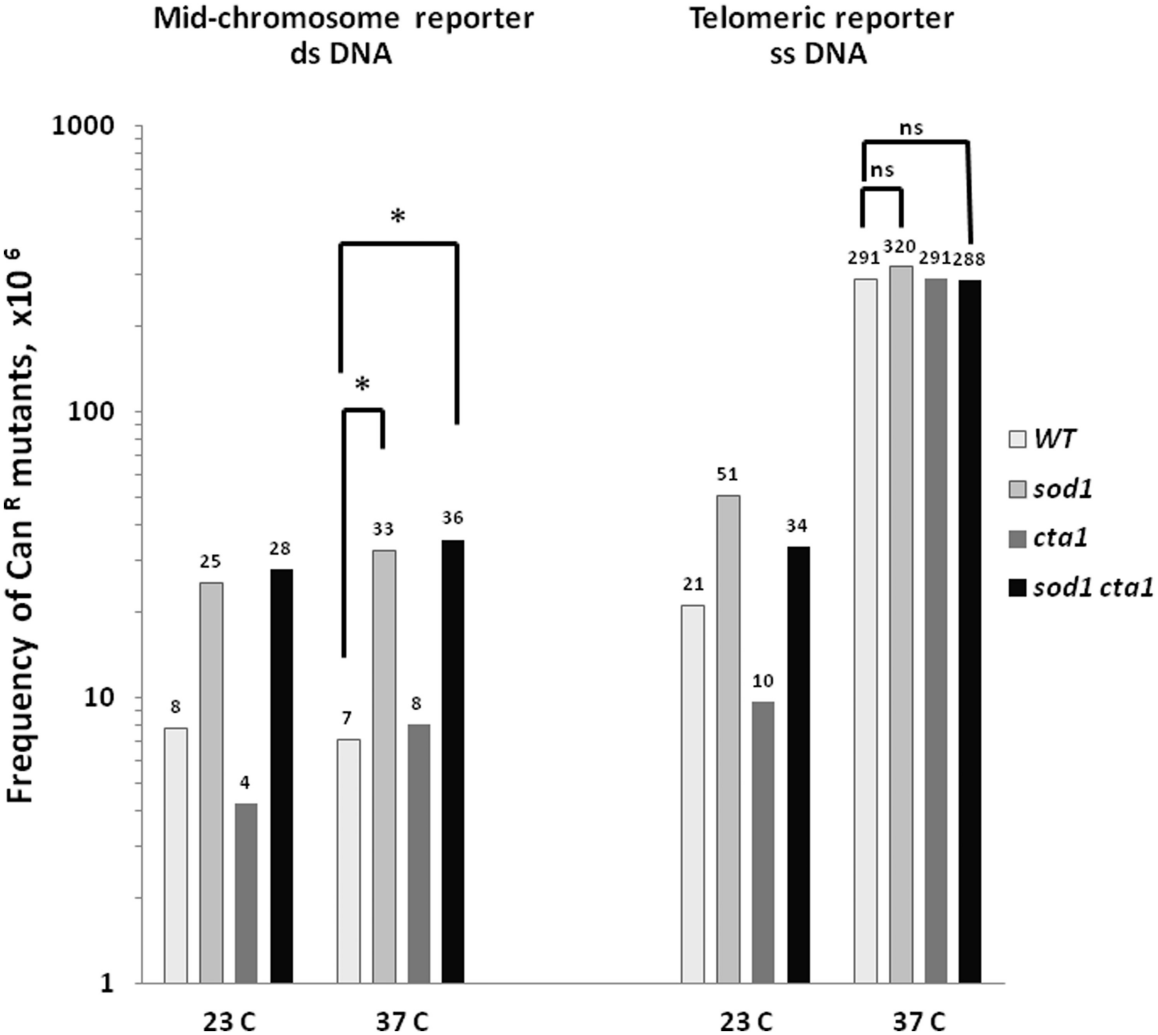
Frequencies of spontaneous mutations in the *CAN1* locus in strains with compromised ROS-scavenging activities. Mutation frequencies at the *CAN1* locus were calculated as described in Materials and Methods section. Average mutation frequency measured for at least six independent cultures incubated at 37°C for 3.5 h to induce telomere un-capping and at 23°C in control experiments. Asterisk indicates statistically significant difference (*P* < 0.005) in frequencies of mutations as determined by Mann–Whitney test. NS indicates the absence of significant differences.

To address the contribution of endogenous ROS to hyper-mutability of ssDNA regions, we first measured the frequencies of mutations in the *CAN1* locus in the strains with decreased ROS-scavenging capacities. Superoxide dismutases and catalases play an important role in oxygen radical and hydrogen peroxide detoxification, respectively (reviewed in (31)). Inactivation of yeast Cta1 catalase or Sod1 superoxide dismutase leads to elevation in endogenous ROS levels (32) and to an increase in mutation rates in a dsDNA reporter (33). Surprisingly, disruption of *SOD1* and/or *CTA1* genes, did not result in a change in mutation frequencies in ssDNA (Figure 2). At the same time mutation frequencies for the mid-chromosome dsDNA reporter in *sod1Δ* mutants were significantly higher than in the wild-type strain (*P* = 0.002) at 37°C. We conclude that the major source of spontaneous mutagenesis in ssDNA is not endogenous ROS, even in mutants with decreased capacity of ROS scavenging. The lack of impact of the endogenous ROS level could be due to effectiveness and redundancy of intracellular ROS-scavenging systems and/or to ssDNA-specific mechanisms of protection against ROS within the cells. Alternatively, it is possible that ROS sub-species produced in ROS-scavenging defective strains, while mutagenic for dsDNA, has no effect on ssDNA. Also, the contribution of endogenous ROS to ssDNA mutagenesis could be over-shadowed by the high levels of alternative sources of spontaneous hyper-mutability in ssDNA, such as decreased fidelity of DNA synthesis during restoration to dsDNA state.

Even though it is unlikely that DNA glycosylases could efficiently remove oxidative lesions in ssDNA substrates (with the exception of uracil-DNA glycosylases (9,34)), and nucleotide incorporation across the abasic sites created by glycosylases would be often mutagenic, we sought to exclude the possibility that DNA glycosylases dealing with oxidative damage in dsDNA are also involved in protection of ssDNA against spontaneous mutagenesis. Spontaneous mutation frequencies in ssDNA reporter were determined in *ogg1Δ*, *ntg1Δ*, *ntg2Δ* and *ntg1Δ ntg2Δ* backgrounds. The ranges of mutation frequencies in wt strains (1.6 − 2.3 × 10^−^^4^) did not differ from the strains with glycosylase(s) knockouts (2.1 − 3.2 × 10^−^^4^). Thus, we confirmed that these DNA glycosylases are not involved in repair of endogenous lesions in ssDNA.

**Figure 3.**
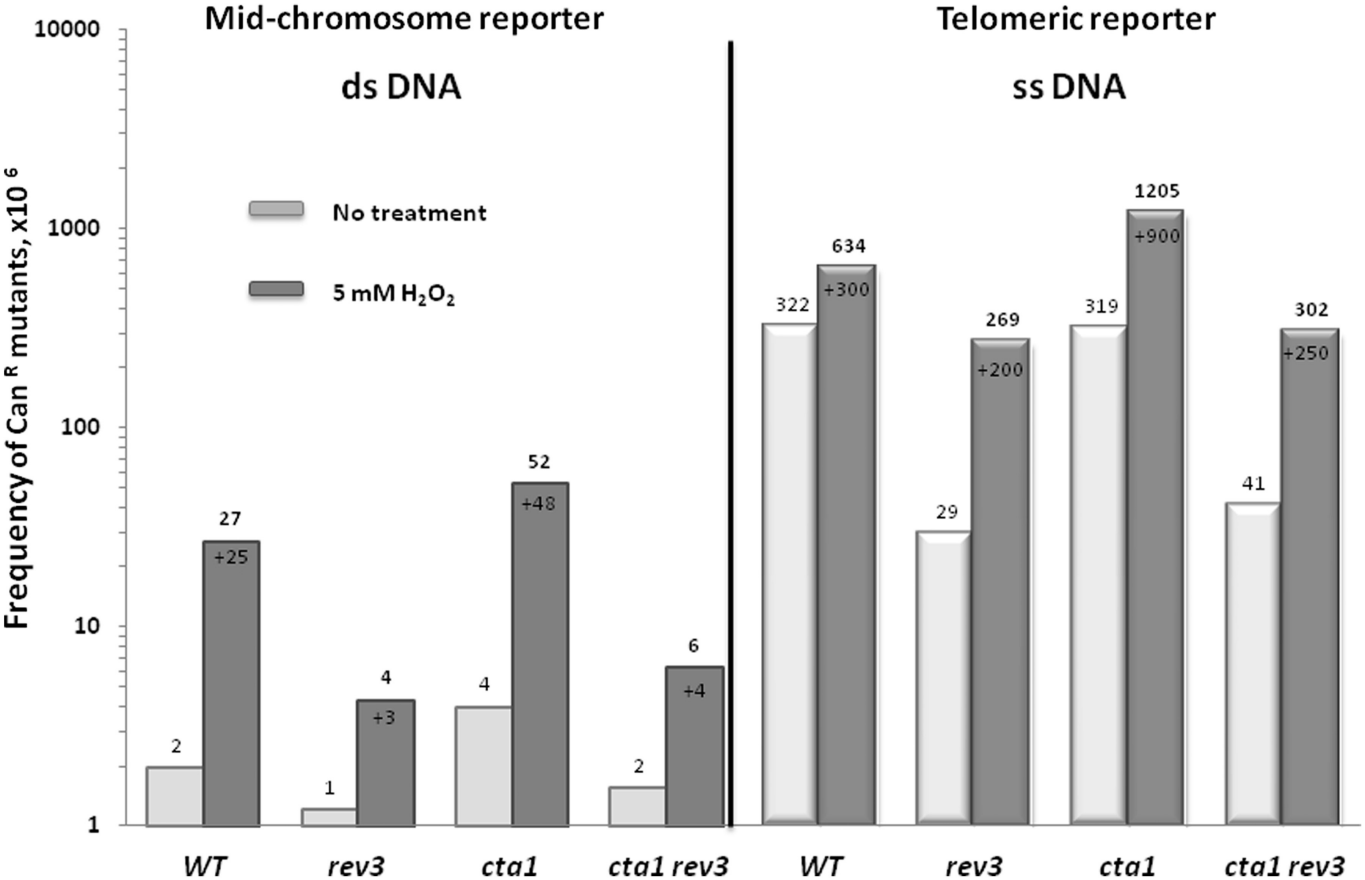
Exogenous oxidative damage induces mutagenesis in ssDNA. Mutation frequencies at the *CAN1* locus were calculated as described in Materials and Methods section. Average mutation frequency measured for at least four independent cultures incubated at 37°C for 3.5 h induce telomere uncapping followed by exposure to 5 mM hydrogen peroxide or mock treatment are presented. Mid-chromosome reporter (described in Results section) was used as a control under the same experimental conditions. Numbers above the bars reflect the absolute frequency of mutation, numbers with ‘+’ indicate how many additional mutations were deposited at *CAN1* locus following exposure to hydrogen peroxide. Standard deviations of average are presented in Supplementary Table S4.

Acute exposure of wild-type cells to 5 mM hydrogen peroxide resulted in only a 2-fold increase in mutation frequency in the ssDNA reporter (Figure 3). However, such exposure caused an ∼300 × 10^6^ absolute increase in mutation frequency superimposed on the already high frequency of spontaneous mutations in the ssDNA reporter within the wild-type strain. This is 12-fold greater than the mutation frequency added by hydrogen peroxide in the dsDNA mid-chromosome reporter. Although the relative increase in mutation frequencies by hydrogen peroxide in a sub-telomeric reporter of wild-type cells was modest, exposure to the same dose of hydrogen peroxide caused a detectable increase in mutation frequency in dsDNA (mid-chromosome reporter), indicating that the chosen dose was indeed mutagenic to chromosomal DNA.

Reducing the cellular capacity to scavenge hydrogen peroxide by deletion of *CTA1* resulted in a statistically significant increase in hydrogen peroxide-induced mutation frequency in the ssDNA reporter, without influencing cell survival (average survival ∼92%). Thus, exogenous oxidative damage causes an increase in the ssDNA mutation frequencies, and cells with compromised ROS-scavenging capacity are sensitized to exogenous ROS-induced mutagenesis.

To eliminate the possibility that sensitivity of ssDNA to an oxidative DNA-damaging agent was a unique effect exerted by hydrogen peroxide, we tested the mutagenicity of another oxidative agent, paraquat. The precise mechanism of paraquat cytotoxicity is not completely understood, but is believed to involve an increase in cellular levels of superoxide anion (35). In agreement with the results for hydrogen peroxide-induced ssDNA mutagenesis, a significant increase in mutagenesis by paraquat was observed in *sod1Δ* strains, known to be highly sensitive to this chemical. However, survival and mutagenesis were essentially unchanged in a wild-type strain (Supplementary Figure S1).

Based on the outcome of the experiments involving two different oxidative DNA-damaging agents we conclude that even though oxidative stress can increase the frequency of mutations in ssDNA, the contribution of oxidative DNA damage to ssDNA mutagenesis is obscured by very high levels of spontaneous mutagenesis originating from other sources. These data and the lack of increase in mutation frequencies in ROS-scavenging mutants suggest that intracellular ROS are not the major source of spontaneous hyper-mutability of ssDNA.

### TLS contributes to ROS-induced ssDNA mutagenesis

**Figure 4.**
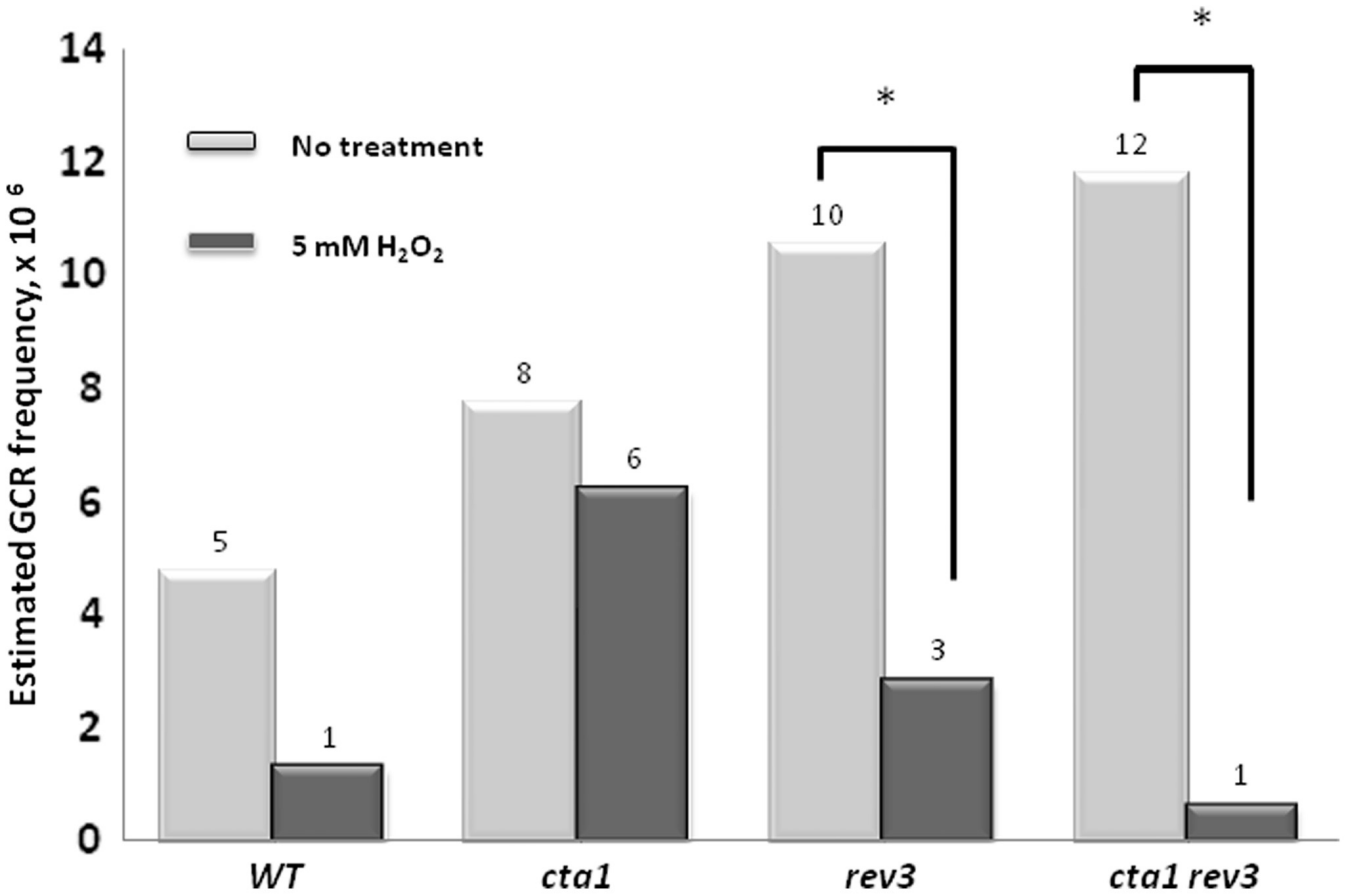
Estimated frequencies of spontaneous and oxidative stress-induced GCRs. Frequencies of GCRs were calculated as the ratio of the number of Can^R^ colonies unable to grow on the media lacking uracil to the total number of cells in the culture. Median frequency of Ura^−^ Can^R^ colonies per genotype per experimental condition for at least four independently selected cultures is presented. Asterisk indicates statistically significant differences (Mann–Whitney test, *P* < 0.03).

The *CAN1-URA3* reporter for ssDNA mutagenesis measurements utilized in this study allows for a direct, forward selection of Can^R^ Ura^−^ cells. Such mutants could arise either as a consequence of independent mutations in both genes or due to the loss of the entire arm of chromosome V between the telomere and the *CAN1* open reading frame. To distinguish between these two mutational outcomes we used a diagnostic PCR with primers specific for the telomere-proximal *URA3* locus in the ssDNA reporter (Materials and Methods section). Our experiments with glycosylase knock-out mutants demonstrated that mutations in ssDNA (unlike in dsDNA) cannot be prevented by BER (see above) and therefore, the spectrum and frequencies of base substitutions and small indels in the ssDNA reporter allows unambiguous assessment of the site and the frequency of occurrence of pre-mutagenic lesions (at least for lesions which cannot be bypassed without an error during the re-synthesis of dsDNA). In contrast, assessment of potential GCR events is not as clear. GCR events could be avoided in ssDNA as well as in dsDNA by template switching to the available sister chromatid during re-synthesis of the resected sub-telomeric region. Also, the unknown fraction of cells in which TLS failed may not survive rather than generate GCR. Thus, the frequency of GCR would be defined by the efficiency of a TLS alternative pathway as well as by other confounding factors. We found that the overall frequency of spontaneous GCRs in *rev3Δ* strains did not increase significantly (Figure 4). Surprisingly, induction of oxidative damage by hydrogen peroxide in *rev3Δ* and *rev3Δ cta1Δ* double mutants substantially decreased the frequency of GCRs. Additional studies are required to address the mechanisms of such a decrease.

We noticed that the fraction of GCRs among spontaneous Can^R^ clones in *rev3Δ* mutants was greater than in wild-type cells (Supplementary Figure S2), suggesting that the decrease in point mutation frequencies and not in GCRs is the major cause of overall lower Can^R^ mutation rates in *rev3Δ* strains. Indeed, in contrast to the negligible contribution of TLS to GCR, elimination of TLS DNA Pol zeta significantly reduced frequencies of ROS-induced point mutations (measured as the frequency of single Can^R^ mutants) in all backgrounds (Figure 3 and Supplementary Figure S1). These results can be explained by ROS causing mutagenic lesions in ssDNA. Alternatively, it is possible that in the context of ssDNA, Pol zeta as well as the replicative polymerases display a higher error propensity under the conditions of oxidative stress (see Discussion section)

### Low mutation density in mutation clusters caused by ROS

The density of mutations in clusters induced by moderately toxic doses of UV, MMS and sulfites in ssDNA in the vicinity of DSBs or at uncapped telomeres is several orders of magnitude higher than the mutation density in dsDNA (6,8,9). Because such different types of DNA damage elicit closely spaced mutations, we sought to determine whether oxidative damage also induces clustered mutations in ssDNA. Since exogenous ROS significantly contribute to the increase in ssDNA mutagenesis in strains deficient for ROS-scavenging, we decided to analyse the sequences of Can^R^ Ura^−^ double mutants arising in *cta1Δ* and *sod1Δ* strains following hydrogen peroxide and paraquat exposure, respectively (Table 1). We reasoned that Can^R^ Ura^−^ clones with double or even multiple mutations within the two open reading frames most likely arise from cells in which 5′ to 3′ resection encompassed the entire sub-telomeric region, including the *CAN1* and *URA3* open reading frames, i.e. from cells where a long stretch of ssDNA had been exposed to the damaging agents. Importantly, when no oxidative agent was applied, PCR analysis of the *CAN1-URA3* locus (Materials and Methods section) of the Can^R^ Ura^−^ clones revealed that they all arose as a consequence of GCR; none were caused by multiple point mutations.

**Table 1.** Density of oxidative agent-induced mutations in *CAN1-URA3* ssDNA reporter

		Number of mutations per *CAN1-URA3* reporter					
Oxidative agent	Strain background				Number of mutants	Total number of mutations	D (mutations per kb)
		2	3	4			
Peroxide	*cta1Δ*	23	3	0	26	55	0.04
Paraquat	*sod1Δ*	20	0	0	20	40	n.d

Surprisingly, only 3 out of 26 of peroxide- and 0 of 20 paraquat-induced Can^R^ Ura^−^ clones in *cta1Δ* and *sod1Δ* backgrounds, respectively, contained more than a single mutation in each of the *CAN1* and *URA3* open reading frames. None of the Can^R^ Ura^−^ clones contained four or more mutations. Such low multiplicity of mutations differed greatly from the density of the mutations reported for UV-induced damage in the *lys2* reporter system (1,6), which is comparable in size to the *CAN1-URA3* reporter employed in our study. Up to six independent mutations were found in the *LYS2* locus and more than 30% of the Lys^-^ clones contained more than two lesions. The reported mutation density for the wild-type strain in those experiments was 0.31 mut/kb (1), whereas the mutation density of oxidative stress-induced mutations in the *CAN1-URA3* reporter in sensitized *cta1Δ* mutants was as low as 0.04 mut/kb (density of unselected mutations calculated as in (1) and ref. therein). Incubation of *sod1Δ* mutants with 150 µM paraquat increased the mutation frequencies 30-fold and decreased cell survival to ∼4% (Supplementary Figure S1); nevertheless, none of the sequenced Can^R^ Ura^−^ clones contained more than two mutations. Importantly, treatment with paraquat resulted in mutation frequencies comparable to those caused by MMS in (8) and paraquat-induced cell killing was greater than the lethality caused by MMS. Additional biochemical and genetic studies are required to understand the difference in mutation density within clusters caused by ROS compared to those caused by alkylation (MMS), deamination (sulfites) or bulky (UV) lesions in DNA (see Discussion section)

### TLS-independent mutations of cytosines are the major source of mutagenesis by hydrogen peroxide- and paraquat-induced oxidative stress in ssDNA

Determining the mutagenic targets of a specific DNA-damaging agent *in vivo* generally relies on analysis of the mutation spectrum. The use of traditional dsDNA reporter systems to assign mutagenic lesions to certain nucleotides is problematic, because any given mutational change originated in dsDNA could be caused by the damage of either nucleotide in a pair. The deduction of nucleotide lesion spectra based on mutation spectra in dsDNA reporters is further complicated by functional DNA repair systems, which may disproportionally repair lesions in different bases and sequence context. The ssDNA reporter system in this study enabled direct identification of the mutated nucleotides, without interference from BER and NER. A lesion can be unambiguously assigned to one of the two nucleotides within a base pair because the 5′ to 3′ direction of the DNA strand resection in the reporter is known. This defines which DNA strand is retained following resection and which nucleotide within a base pair is the target for damage or was erroneously copied by a DNA polymerase.

**Figure 5.**
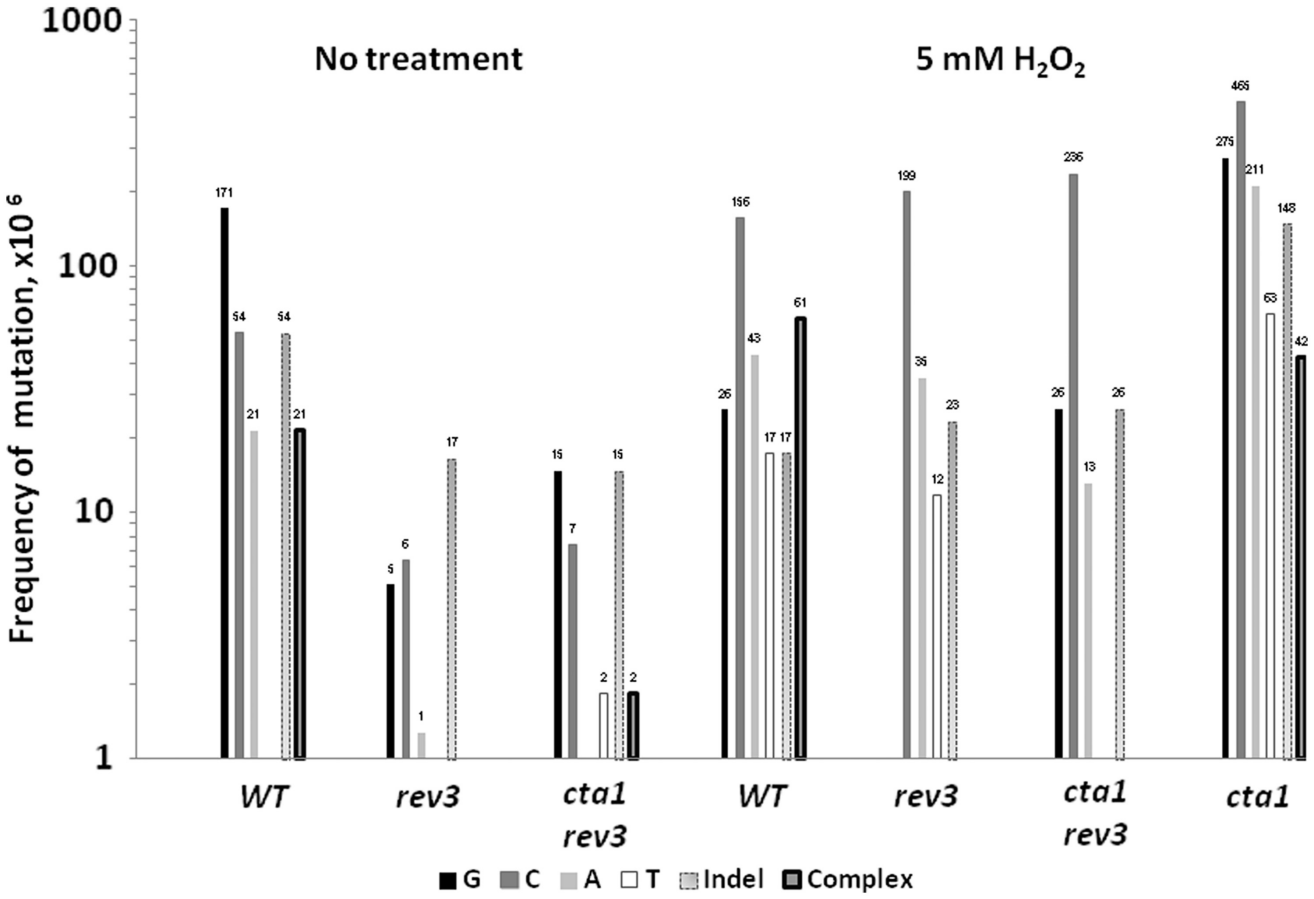
Frequencies of endogenous and oxidative damage-induced substitutions, complex mutations and insertions/deletions calculated by multiplication of the fraction of each type of mutation (Supplementary Table S1) to the total frequency of spontaneous mutations or following exposure to 5 mM hydrogen peroxide in the strains of different genetic backgrounds.

We calculated the frequencies of different types of mutation events (Figure 5) by multiplying the total mutation frequency by the fraction of the specific type of the mutation event within the mutation spectrum (Supplementary Table S1). Comparison of *spontaneous* mutation spectra in the *CAN1* locus between *rev3Δ* and *REV3* strains indicated that error-prone bypass of guanines is the greatest source of mutations in the *REV3* strain (Figure 5, ‘no treatment’ panel). In a *REV3* strain mutations at G occur ∼30- and ∼10-fold more frequently than in *rev3Δ CTA1* and *rev3Δ cta1Δ* strains, respectively· Insertions of A across positions of G in ssDNA occurred most often, since G to T transversions constituted the majority of substitutions of guanine (Supplementary Table S2). DNA polymerase zeta was also responsible for 90–95% of spontaneous substitution frequencies of A and C (Figure 5). The contribution of substitutions of T to spontaneous mutagenesis was negligible in all genotypes. Presently, it is not possible to ascertain whether DNA polymerase zeta erroneously bypasses endogenously damaged nucleotides or whether the mutations are caused by mutagenic bypass of undamaged nucleotides due to a proposed defective replisome-induced mutagenesis mechanism (36).

In the analyses of mutations *induced by oxidative agents* (hydrogen peroxide in wild type and in *cta1Δ* and paraquat in *sod1Δ* strains, respectively), we included only mutations from isolates with simultaneous inactivation of *CAN1* and *URA3*, because those were likely to have originated from lesions in ssDNA, as discussed above. In ROS-scavenging proficient strains, the majority of Can^R^ Ura^−^ clones arose from GCR events whereas ssDNA double mutants were observed at low frequencies. We reasoned that since in all backgrounds there was ≥10-fold increase in mutation frequencies in the sub-telomeric ssDNA reporter as compared to mid-chromosome (dsDNA) reporter, all the Can^R^ mutants, including Can^R^ Ura^+^ have originated from independent mutagenesis events in ssDNA of non-dividing cells. In striking contrast to the spontaneous mutation spectra, base substitutions at cytosines in the putative ssDNA strand comprised the largest part of mutations in all the strains subjected to oxidative stress (Supplementary Table S1).

To test the possibility that the burden of 8oxoG accumulated in ssDNA following oxidative stress could be obscured by error-free bypass of 8oxoG by TLS DNA polymerase eta, we measured the frequencies of Can^R^ mutations in *rad30Δ* strains (Supplementary Table S3). Frequencies of the oxidative stress-induced mutations in *cta1Δ* and *cta1Δ rad30Δ* strains, as well as in wt and *rad30Δ* were not significantly different (Mann–Whitney test *P* = 0.44 and 0.65, respectively). Thus, since Ogg1 does not remove 8oxoG from ssDNA (see above) it is unlikely that hydrogen peroxide induce significant number of lesions at G in ssDNA.

Incorporation of oxidized nucleotides during DNA replication causes mutations in prokaryotic and eukaryotic organisms (37,38). An increase in A–C and G–T transversions would be indicative of incorporation of 8oxodG across A and 2-OH-dA across G, respectively, in ssDNA under oxidative stress conditions. Since mutations at A are less frequent than at C and G (Figure 5) and G–T transversions is not the major class of oxidative stress-induced mutations at G (Supplementary Table S2), it is unlikely that that insertion of oxidized nucleotides during the re-synthesis of the second strand of DNA contributes significantly to oxidative stress-induced mutagenesis in ssDNA.

In summary, *oxidative* damage-induced mutations occurred with the highest frequency at cytosines. This differed from different from *spontaneous* mutations, where the most frequent events were indels or mutations at guanines. The proportion of mutations at C among all mutations under oxidative stress conditions was significantly higher compared to that for spontaneous mutagenesis (*P* < 0.0001; Fisher’s exact test).

We then explored the role of Pol zeta TLS in ssDNA mutagenesis caused by *oxidative* stress. In agreement with other reports (36,39) the Rev3 deletion caused a strong decrease in frequencies of hydrogen peroxide-induced complex mutations (Figure 5). We also noticed that the effect of Rev3 deletion on oxidative mutagenesis in ssDNA was moderate (2.4-fold decrease in the mutation frequencies in *rev3Δ* strain) as compared to the effects on mutagenesis caused by UV, MMS and sulfites (6,8,9). The *rev3Δ* mutants retained ∼25% of ssDNA oxidative stress-induced mutagenesis in *cta1Δ* or in *sod1Δ* and even 42% in a wild-type background (Figure 3 and Supplementary Figure S1). In contrast to other types of DNA lesions, the frequency of base substitutions at cytosines did not decrease in the absence of Rev3 (Figure 5), suggesting that the mutator effect of damage to cytosines or their erroneous bypass caused by oxidative stress conditions is Rev3 independent. The reasonable candidate for such a lesion would be deaminated cytosine creating uracil (U) in ssDNA. If U remains in ssDNA until second-strand DNA synthesis, it would lead to C to T mutations, which are prevalent in all three mutation spectra (hydrogen peroxide-induced in wild type and *cta1Δ* and paraquat-induced in *sod1Δ*). Since uracil in DNA is as good a template for A pairing as a T, such mutations would be TLS independent. However, it was previously established that U, which was created in ssDNA *in vivo* through enzymatic deamination by endogenously expressed APOBEC3G appears to be a good substrate for uracil-DNA glycosylase (Ung1). The resulting mutations were approximately equal for C–T (45%) and C–G (51%) with the small fraction of C–A (3%) (9). In our study distribution of C changes caused by oxidative agents in wild type, *cta1Δ* and *sod1Δ* (C–T—56%, C–G—22%, C–A—22%) clearly differed from the distribution obtained with APOBEC3G enzymatic cytosine deamination (*P* = 0.005, chi-square test). If the major mutagenic lesions at C arise as a consequence of oxidative damage-induced C–U deamination, deletion of Ung1 should have resulted in an increase of C–T transitions. Importantly, we did not observe such increase in *ung1Δ* strains (Supplementary Table S2). The distribution of hydrogen peroxide-induced changes at C in the *ung1*Δ strain (C–T—50%, C–G—20%, C–A—30%) did not differ from that of wild-type strain (*P* = 0.8, chi-square test). These results indicate that C deamination is not the major mutagenic event in ssDNA, and that different type(s) of ROS-induced DNA lesions may contribute significantly to mutagenesis at C (see ‘Discussion’ section).

## DISCUSSION

### Oxidative stress and mutability of ssDNA

Recently, hyper-mutability of ssDNA and its potential relevance to cancer was established (2,3). Analysis of genome-wide mutation datasets from four types of human cancers identified strand-coordinated clusters of mutations, often occurring in the vicinity of the genome rearrangement breakpoints. Even though the precise mechanism of generation of such clusters is largely unknown, clusters as well as single mutation events were significantly enriched for the mutation motifs characteristic for the APOBEC family of cytosine deaminases, mutator enzymes highly preferring ssDNA to dsDNA, which indicated that there is a sufficient amount of ssDNA to account for a sizable fraction of mutagenesis in cancers. It appeared plausible that physiologically relevant, persistent increases in ROS levels caused by chronic inflammation and/or defective energy metabolism in cancer cells or a transient redox imbalance due to signal transduction processes could also contribute to mutagenesis of ssDNA.

Utilizing yeast as model system we found that, in contrast to our expectations, an increase in endogenous levels of ROS caused by defined genetic defects did not mutagenize ssDNA. Importantly, mutants caused by ROS-generating treatments had a much lower density of mutations as compared to MMS-, UV- and sulfite-induced mutants, which often contained clusters of multiple mutations. These findings suggest the existence of either an unknown pathway of repairing BER substrates in ssDNA or specialized mechanisms for ssDNA protection against oxidative damage. Also, it is possible that ssDNA at uncapped telomeres is protected from oxidative, but not from UV- and MMS-induced damage by telomere-specific ssDNA binding proteins. Although exposure to exogenous oxidative agents increased the mutability of ssDNA it is clear that high spontaneous levels of ssDNA mutagenesis are caused by sources different from endogenous ROS. Nevertheless, even though the consequences of exposure to oxidative DNA-damaging agents were less relevant to localized hyper-mutability compared to the effects of MMS-induced alkylating damage and UV-induced damage, compromising the endogenous cellular antioxidant defenses significantly increased the frequency of ROS-induced mutations in ssDNA.

The above findings could have important translational implications. Several lines of evidence suggest that altered metabolism of cancer cells places them under continuous ROS stress due to the Warburg effect (40). Exposure of cancer cells to chronic, endogenous oxidative stress could exploit the higher vulnerability of cancer cells to DNA damage-inducing therapeutic agents [reviewed in (41)]. It was shown recently that a small molecule targeting ROS response can cause selective killing of cancer cells independently of their p53 status (42). Our results suggest that therapies aimed at diminishing activities of superoxide dismutase or catalase, could selectively increase the mutational load in ssDNA of actively replicating cancer cells, overloaded with ROS. Such increased mutational pressure could lead to cell death due to multiple mutations in essential genes or via apoptosis caused by activation of replicative checkpoints. In contrast, increasing the endogenous levels of ROS in normal tissues by suppressing antioxidant defenses should not cause deleterious effects.

### Cytosines are the major source of ROS-induced mutagenesis in ssDNA

8-Oxoguanine is thought to be the most frequently occurring DNA lesion caused by oxidation (summarized in (43)). However, it remains unclear whether this lesion is most mutagenic since analyses of mutation spectra indicate that C–T transitions are prevalent among spontaneous base substitutions *in vivo* (44,45). In order to address the mutational potential of different nucleotides under oxidative stress conditions we utilized a reporter in which precise identity of the pre-mutagenic lesions is not obscured by action of DNA excision repair systems. It also allowed discrimination between two bases within a mutated base pair, because the strand that is being resected during the telomere-uncapping step is known. These features of the ssDNA reporter system revealed that cytosines are the major source of oxidative damage-induced mutations in ssDNA and implicate cytosine as the major source of the mutations in a GC pair. Our findings raise several important questions. Are cytosine modifications the most common oxidative DNA lesions, and are they the most mutagenic or both? It is possible that oxidative deamination is the major source of mutagenesis at C under oxidative stress conditions, even though the fraction of C–T substitutions among all C substitutions is much higher compared to conditions when, in the presence of Ung1, APOBEC3G-induced deamination produces almost equal numbers of C–T and C–G substitutions. Accordingly, if C deamination is not the only source of mutations at C, what types of oxidative cytosine modification prompt mis-pairing with dATP by the replicative DNA polymerases? It has been shown that products of cytosine oxidation and deamination, namely, 5-hydroxyuracil and uracil glycol, efficiently cause C–T substitutions *in vivo* when single-stranded oligos, containing such modified bases are replicated in *Escherichia coli* (46). 5-Hydroxycytosine, a product of direct oxidation of cytosine, exhibits weaker mutagenic properties. Its miscoding properties are dictated by instability in the replicative polymerase active site, which allows dATP misincorporation in a process similar to that described for incorporation opposite to abasic sites (47). In addition, chemical interaction of dCTP with oxidants generates 5-hydroxycytidine, which can be incorporated into DNA and is highly mutagenic when bypassed by *E. coli* Pol I (48). Taken together, these results suggest that the products of oxidation and/or deamination of cytosine are important genome-destabilizing products of oxidative damage *in vivo*. Development of analytical methods for rapid detection of such modified nucleotides should provide precise and meaningful measurements of endogenous oxidative stress.

Among the four common DNA bases, cytosines were found to be mutated most frequently following exposure to exogenous oxidative damage. Nevertheless, the majority of *spontaneous* mutations were caused by error-prone, DNA polymerase zeta-mediated bypass by of guanines. These observations allow us to speculate that endogenously—produced ROS may not be the major contributors to spontaneous mutagenesis or that exogenous oxidative agents are metabolized in the cells to ROS sub-types that are different from those produced endogenously and cause a different spectrum of mutagenic base modifications. It is important to note that it is possible that the high mutagenic potential of C under oxidative stress results from a greater propensity for replicative DNA polymerases to incorporate erroneous nucleotides across non-damaged C in ssDNA in a highly oxidative intracellular environment.

**Figure 6.**
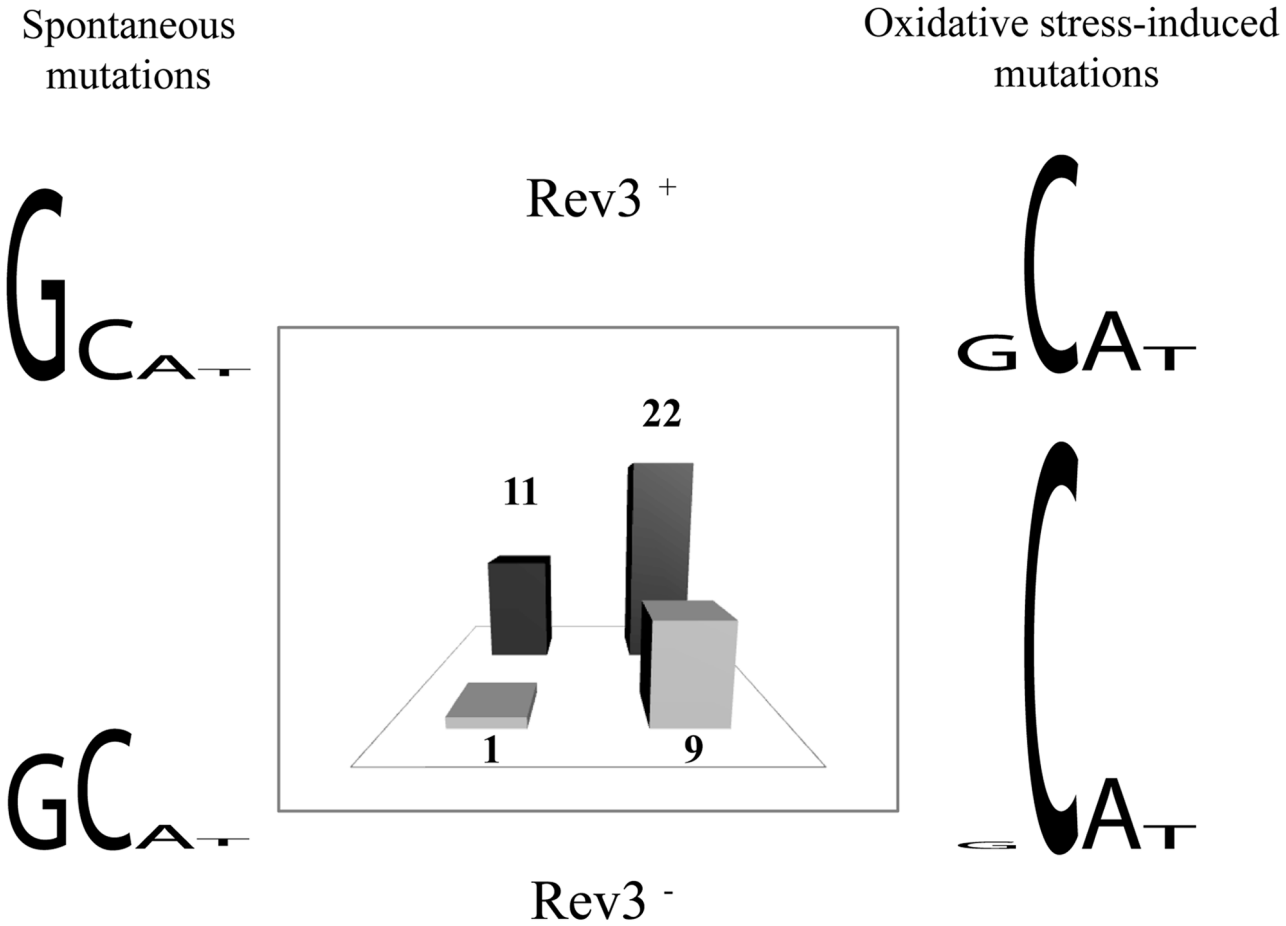
Mutagenic potential of the nucleotides and role of TLS Pol zeta in ROS-induced mutagenesis. Propensity of the pre-mutagenic lesions to cause spontaneous and oxidative stress-induced mutations and the role of TLS in the mutagenesis is schematically depicted. Fraction of the mutations occurred at each nucleotide calculated from Supplementary Table S1 for each experimental condition. The height of corresponding letter is proportional to the contribution of each nucleotide to ssDNA mutagenesis. Bar graph reflects the relative frequencies of mutations at *CAN1* locus in ssDNA reporter for each experimental condition in wt (Rev3+) and *rev3Δ* (Rev3−) strains.

In summary, our study of ROS-induced ssDNA mutagenesis suggests that ROS are not the major source of spontaneous ssDNA mutagenesis and that TLS polymerase zeta contributes significantly to both *ROS-induced* and *spontaneous* ssDNA mutagenesis. Under *oxidative* stress, C is the most mutagenic nucleotide (Figure 6). Its mutagenic potential is TLS independent, whereas s*pontaneous* base substitutions in ssDNA occur mainly due to DNA polymerase zeta-dependent mutagenic bypass of guanines.

## SUPPLEMENTARY DATA

Supplementary Data are available at NAR Online.

## FUNDING

Funding for open access charge: National Institute of Environmental Health Sciences [ES011163 to P.W.D.]; Intramural Research Program of the National Institute of Environmental Health Sciences [ES065073 to M.A.R.].

*Conflict of interest statement*. None declared.

## Supplementary Material

Supplementary Data
