# Supplementary material for: Oxidative stress-induced mutagenesis in single-strand DNA occurs primarily at cytosines and is DNA polymerase zeta-dependent only for adenines and guanines
Source: Nucleic Acids Res. 2013 Aug 7;41(19):8995–9005. doi: 10.1093/nar/gkt671 (PMC3799438; doi:10.1093/nar/gkt671)
Supplement: Supplementary Data [file supp_41_19_8995__index.html]

Oxidative stress-induced mutagenesis in single-strand DNA occurs primarily at cytosines and is DNA polymerase zeta-dependent only for adenines and guanines — Oxidative stress-induced mutagenesis in single-strand DNA occurs primarily at cytosines and is DNA polymerase zeta-dependent only for adenines and guanines — Supplementary Data 

# Oxidative stress-induced mutagenesis in single-strand DNA occurs primarily at cytosines and is DNA polymerase zeta-dependent only for adenines and guanines

## 

files

**Files in this Data Supplement:**

- Supplementary Data - pdf file
- Supplementary Data - pdf file
