# Supplementary material for: Oxidative stress-induced mutagenesis in single-strand DNA occurs primarily at cytosines and is DNA polymerase zeta-dependent only for adenines and guanines
Source: Nucleic Acids Res. 2013 Aug 7;41(19):8995–9005. doi: 10.1093/nar/gkt671 (PMC3799438; doi:10.1093/nar/gkt671)
Supplement: Supplementary Data [file supp_gkt671_nar-01149-f-2013-File010.pdf]

mutant strain	relevant genotype	spontaneous or induced?	mutated gene	position in Chr. V (distance from telomere)	WT base(s)
LHY63	WT	5mM H_2O_2	*URA3*	3800	TCA
			CAN1	6132	T
LHY64	WT	5mM H_2O_2	*URA3*	4220	G
			CAN1	5984	G
LHY65	WT	5mM H_2O_2	*URA3*	3874	AC
			CAN1	6615	GG
LHY66	WT	5mM H_2O_2	*URA3*	4141	G
			CAN1	6120	G
LHY67	WT	5mM H_2O_2	*URA3*	3544	C
			CAN1	6149	G
LHY68	WT	5mM H_2O_2	*URA3*	3543	TC
			URA3	3571	G
			CAN1	5895	G
LHY69	WT	5mM H_2O_2	*URA3*	4123	G
			CAN1	5621	G
LHY70	WT	5mM H_2O_2	*URA3*	3599	G
			CAN1	5804	TTCAAT
LHY71	WT	5mM H_2O_2	*URA3*	3898	G
			CAN1	6769	CAC
LHY72	WT	5mM H_2O_2	*URA3*	4028	T
			CAN1	5893	C
LHY73	WT	5mM H_2O_2	*URA3*	4171	A
			CAN1	6292	C
LHY74	WT	5mM H_2O_2	*URA3*	3559	T
			CAN1	5754	G
LHY104	WT	5mM H_2O_2	*URA3*	4208	G
			CAN1	5471	G
LHY107	WT	5mM H_2O_2	*URA3*	3602	G
			CAN1	5733	G
LHY108	WT	5mM H_2O_2	*URA3*	4081	A
			CAN1	5481	G
LHY109	WT	5mM H_2O_2	*URA3*	3953	C
			CAN1	5576	G
LHY115	WT	5mM H_2O_2	*URA3*	4061	CATGTG
			CAN1	6119	G
LHY117	WT	5mM H_2O_2	*URA3*	3812	T
			CAN1	6657	T

LHY75	*cta1Δ*	5mM H_2O_2	*URA3*	4220	G
			CAN1	5634	C
LHY76	*cta1Δ*	5mM H_2O_2	*URA3*	4271	C
			CAN1	5721	G
LHY77	*cta1Δ*	5mM H_2O_2	*URA3*	3694	G
			CAN1	6653	G
LHY79	*cta1Δ*	5mM H_2O_2	*URA3*	3793	G
			CAN1	5772	T
LHY80	*cta1Δ*	5mM H_2O_2	*URA3*	3644	T
			URA3	3662	A
			CAN1	5679	C
LHY81	*cta1Δ*	5mM H_2O_2	*URA3*	3659	C
			CAN1	6119	G
LHY82	*cta1Δ*	5mM H_2O_2	*URA3*	3638	C
			CAN1	6618	T
LHY83	*cta1Δ*	5mM H_2O_2	*URA3*	3830	C
			CAN1	6029	T
LHY84	*cta1Δ*	5mM H_2O_2	*URA3*	3602	G
			CAN1	5774	G
LHY85	*cta1Δ*	5mM H_2O_2	*URA3*	4123	G
			CAN1	5450	G
LHY86	*cta1Δ*	5mM H_2O_2	*URA3*	3790	TCTG
			CAN1	5882	G
LHY87	*cta1Δ*	5mM H_2O_2	*URA3*	3545	C
			CAN1	5999	G
LHY88	*cta1Δ*	5mM H_2O_2	*URA3*	4064	G
			CAN1	6364	TGTT
LHY89	*cta1Δ*	5mM H_2O_2	*URA3*	3490	C
			CAN1	6847	G
LHY 90	*cta1Δ*	5mM H_2O_2	*URA3*	3900	C
LHY121	*cta1Δ*	5mM H_2O_2	*URA3*	3520	C
			CAN1	6664	C
LHY122	*cta1Δ*	5mM H_2O_2	*URA3*	4014	T
			CAN1	5409	A
LHY123	*cta1Δ*	5mM H_2O_2	*URA3*	4000	G
			URA3-CAN1	4259	T
			CAN1	5903	G
LHY124	*cta1Δ*	5mM H_2O_2	*URA3*	3937	A
			CAN1	5586	G
LHY125	*cta1Δ*	5mM H_2O_2	*URA3*	4243	A
			CAN1	6432	G
LHY127	*cta1Δ*	5mM H_2O_2	*URA3*	4156	G
			CAN1	6119	G

LHY129	*cta1 Δ*	5mM H_2O_2	*URA3*	4156	G
			CAN1	6019	delC
LHY133	*cta1 Δ*	5mM H_2O_2	*URA3*	3993	G
			CAN1	5717	T
			CAN1	5743	T
LHY134	*cta1 Δ*	5mM H_2O_2	*URA3*	3563	A
			CAN1	6390	C
LHY135	*cta1 Δ*	5mM H_2O_2	*URA3*	3754	T
			CAN1	5513	G
LHY137	*cta1 Δ*	5mM H_2O_2	*URA3*	3895	T
			CAN1	6082	G
LHY139	*cta1 Δ*	5mM H_2O_2	*URA3*	3595	T
			CAN1	5337	C
LHY221	*sod1 Δ*	150uM paraquat	*URA3*	4040	A
			CAN1	6120	G
LHY222	*sod1 Δ*	150uM paraquat	*URA3*	4214	C
			CAN1	6378	CAACCA
LHY223	*sod1 Δ*	150uM paraquat	*URA3*	3805	A
			CAN1	5667	C
LHY225	*sod1 Δ*	150uM paraquat	*URA3*	3702	-
			CAN1	5739	T
LHY226	*sod1 Δ*	150uM paraquat	*URA3*	3544	C
			CAN1	6111	C
LHY227	*sod1 Δ*	150uM paraquat	*URA3*	3692	G
			CAN1	5461	A
LHY228	*sod1 Δ*	150uM paraquat	*URA3*	3962	C
			CAN1	5746	G
LHY230	*sod1 Δ*	150uM paraquat	*URA3*	4225	C
			CAN1	6204	G
LHY231	*sod1 Δ*	150uM paraquat	*CAN1*	6280	A
			CAN1	6885	G
LHY232	*sod1 Δ*	150uM paraquat	*URA3*	4116	T
			CAN1	6419	C
LHY233	*sod1 Δ*	150uM paraquat	*URA3*	4208	G
			CAN1	5850	G
LHY235	*sod1 Δ*	150uM paraquat	*URA3*	4075	A
			CAN1	5745	G
LHY237	*sod1 Δ*	150uM paraquat	*URA3*	4013	T
			CAN1	6147	G
LHY238	*sod1 Δ*	150uM paraquat	*URA3*	3922	C
			CAN1	5685	C
LHY239	*sod1 Δ*	150uM paraquat	*URA3*	3704	C
			CAN1	5744	T
LHY240	*sod1 Δ*	150uM paraquat	*URA3*	4220	G
			CAN1	5774	G
LHY241	*sod1 Δ*	150uM paraquat	*URA3*	4038	TGG

			CAN1	5965	TAT
LHY242	*sod1 Δ*	150uM paraquat	*URA3*	3482	C
			CAN1	6636	A
LHY243	*sod1 Δ*	150uM paraquat	*URA3*	4077	A
			CAN1	6710	C
LHY244	*sod1 Δ*	150uM paraquat	*URA3*	3982	A
LHY245	*sod1 Δ*	150uM paraquat	*URA3*	3812	T
			CAN1	5850	G

mutant base(s)	deletion/insertion (-/+ number of bases)	5′ flanking sequence	3′ flanking sequence
GCAA		GCATGACAATTCTGCTAACA	AAAGGCCTCTAGGTTCCTTT
A		ATTTCAAGGTACTGAACTAG	TGGTATCACTGCTGGTGAAG
A		ACTAGGATGAGTAGCAGCAC	TTCCTTATATGTAGCTTTCG
-	-1	GTATGGTTTGTGGTGCTGGG	TTACCGGCCCAGTTGGATTC
TA		TAACAATACCTGGACCCACC	ACCGTGTGCATTCGTAATGT
AA		TGTTGCAGGCTTTTTTGCAT	TTATTTATCTCAATCTCGCA
A		TGGTGGTACGAACATCCAAT	AAGCACACAAGTTTGTTTGC
A		CGCTGCCTTCACATTTCAAG	TACTGAACTAGTTGGTATCA
-	-1	CCTTTGCAAATAGTCCTCTT	CAACAATAATAATGTCAGAT
A		TAGTTGGTATCACTGCTGGT	AAGCTGCAAACCCCAGAAAA
AT		CCCTTTGCAAATAGTCCTCT	CAACAATAATAATGTCAGAT
C		TAATAATGTCAGATCCTGTA	AGACCACATCATCCACGGTT
A		GTTCCCTGTCAAATATTACG	TGAATTCGAGTTCTGGGTCG
A		CTAACTCCAGTAATTCCTTG	TGGTACGAACATCCAATGAA
C		ATTTATTTATGGGTTCTTTG	CATATTCTGTCACGCAGTCC
A		ATCATCCACGGTTCTATACT	TTGACCCAATGCGTCTCCCT
T-CAAA		TAGTGTAGTTGGCCAAGTCA	TTTGGACGTACAAAGTTCCA
C		CGTGTGCATTCGTAATGTCT	CCCATTCTGCTATTCTGTAT
AA		ATTATCATTATTCAAGGTTT	GGCTTTTGCACCAAAATTCA
G		TGCCTTTAGCGGCTTAACTG	GCCCTCCATGGAAAAATCAG
G		TTGTTCCCTGTCAAATATTA	GGTGAATTCGAGTTCTGGGT
C		AGTTTGTTTGCTTTTCGTGC	TGATATTAAATAGCTTGGCA
A		CTAACACAATCTACTTCCTA	GTTTCTACTTCTCCCTTTAT
A		CTCTTCCAACAATAATAATG	CAGATCCTGTAGAGACCACA
T		TTACATGTATTGGTTTTCTT	GGCAATCACTTTTGCCCTGG
A		GGCAGCAACAGGACTAGGAT	AGTAGCAGCACGTTCCTTAT
T		AAGGAGAAGTACAGAACGCT	AAGTGAAGAGAGAGCTTAAG
C		ATCCACGGTTCTATACTGTT	ACCCAATGCGTCTCCCTTGT
A		AGCATTTGGTGCGGCCAATG	TTACATGTATTGGTTTTCTT
C		CATGTGTTTTTAGTAAACAA	TTTTGGGACCTAATGCTTCA
A		ACAGAACGCTGAAGTGAAGA	AGAGCTTAAGCAAAGACATA
G		CTGCAATTTGACTGTACTAC	AATGTCAGCAAATTTTCTGT
T		CCACACCTCTGACCAACGCC	GCCCAGTGGGCGCTCTTATA
AAT-TT		AAAATCAGTCAAGATATCCA	TTTTTAGTAAACAAATTTTG
T		ACGCTGCCTTCACATTTCAA	GTACTGAACTAGTTGGTATC
A		TGCTAACATCAAAAGGCCTC	AGGTTCCTTTATTACTTCTT
A		CATCAGATTTATGCAAGCTT	GAAATACCGTGGCATCTCTC

A		ACTAGGATGAGTAGCAGCAC	TTCCTTATATGTAGCTTTCG
G		TTCTTTGGCATATTCTGTCA	GCAGTCCTTGGGCGAAATGG
G		AGGACTAGGATGAGTAGCAG	ACGTTCCTTATATGTAGCTT
A		ATTCCTTTCTCCAGCATTTG	TGCGGCCAATGGTTACATGT
A		TTCCACCCATGTCTCTTTGA	CAATAAAGCCGATAACAAAA
C		CGCACATCAGATTTATGCAA	CTTTGAAATACCGTGGCATC
T		AGCCCTTGCATGACAATTCT	CTAACATCAAAAGGCCTCTA
A		TTGGGCAATCACTTTTGCCC	GGAACTTAGTGTAGTTGGCC
G		ATCTAAACCCACACCGGGTG	CATAATCAACCAATCGTAAC
T		TGTCATAATCAACCAATCGT	ACCTTCATCTCTTCCACCCA
A		ATTCATCCCTGTTACATCCT	TTTCACAGTGTTCTCACAAA
A		GGGTGTCATAATCAACCAAT	GTAACCTTCATCTCTTCCAC
A		ACGCTGCCTTCACATTTCAA	GTACTGAACTAGTTGGTATC
A		CTTGTCATCTAAACCCACAC	GGGTGTCATAATCAACCAAT
A		TGCAGGCTTTTTTGCATGGT	ATTTATCTCAATCTCGCACA
A		TCTAGGTTCCTTTATTACTT	TTCTGCCGCCTGCTTCAAAC
C		ATTGGAGAAACCCAGGTGCC	GGGGTCCAGGTATAATATCT
A		ATCCACGGTTCTATACTGTT	ACCCAATGCGTCTCCCTTGT
A		GGGCAATCACTTTTGCCCTG	AACTTAGTGTAGTTGGCCAA
A		CTAACTCCAGTAATTCCTTG	TGGTACGAACATCCAATGAA
T		ATGGCATAGGTGATGAAGAT	AAGGAGAAGTACAGAACGCT
A-T-		GGGAGCCCTTGCATGACAAT	CTAACATCAAAAGGCCTCTA
T		CAATAATGAACTTGTTCCCT	CAATAATGAACTTGTTCCCT
G		CTTTGCAAATAGTCCTCTTC	AACAATAATAATGTCAGATC
T		CTGGGGTTACCGGCCCAGTT	GATTCCGTTATTGGAGAAAC
C		ATCAGTCAAGATATCCACAT	TGTTTTTAGTAAACAAATTT
-	-4	TTGCCACATATCTTCAACGC	ATCTTAACAACCATTATCTC
A		ATGCTTCCCAGCCTGCTTTT	TGTAACGTTCACCCTCTACC
A		TTCCTGTTCTTAGCTGTTTG	ATCTTATTTCAATGCATATT
A		TGTGCATTCGTAATGTCTGC	CATTCTGCTATTCTGTATAC
A		CACCCTCTACCTTAGCATCC	TTCCCTTTGCAAATAGTCCT
A		TTTATGCAAGCTTTGAAATA	CGTGGCATCTCTCGTGACGA
-	-1	TACTTGGCGGATAATGCCTT	AGCGGCTTAACTGTGCCCTC
-	-1	CCCGACGAGAGTAAATGGCG	GGATACGTTCTCTATGGAGG
-	-1	AGAGTAAAAAATTGTACTTG	AGAGTAAAAAATTGTACTTG
A		CGACATGATTTATCTTCGTT	CCTGCAGGTTTTTGTTCTGT
A		TCAAATATTACGGTGAATTC	AGTTCTGGGTCGCTTCCATC
-	-1	TACACCCGCAGAGTACTGCA	TTTGACTGTACTACCAATGT
T		GACCAACGCCGGCCCAGTGG	CGCTCTTATATCATATTTAT
T		CCTTATATGTAGCTTTCGAC	TGATTTATCTTCGTTTCCTG
A		TGGTTCCCGTATTTTATTTG	TCTATCAAAGAACAAGTTGG
C		CCAATGAAGCACACAAGTTT	TTTGCTTTTCGTGCATGATA
A		ACGCTGCCTTCACATTTCAA	GTACTGAACTAGTTGGTATC

A		CCAATGAAGCACACAAGTTT	TTTGCTTTTCGTGCATGATA
insAA		GGATTCCGTTATTGGAGAAA	CCAGGTGCCTGGGGTCCAGG
C		TCTTCGAAGAGTAAAAAATT	TACTTGGCGGATAATGCCTT
A		AAAGATTCCTTTCTCCAGCA	TTGGTGCGGCCAATGGTTAC
A		GCGGCCAATGGTTACATGTA	TGGTTTTCTTGGGCAATCAC
G		TCCAACAATAATAATGTCAG	TCCTGTAGAGACCACATCAT
A		CTTAACAACCATTATCTCTG	CGCAAATTCAAATATTTACG
-	-1	TGTCAACAGTACCCTTAGTA	ATTCTCCAGTAGATAGGGAG
-	-1	AAAGACATATTGGTATGATT	CCCTTGGTGGTACTATTGGT
C		CACCGTGTGCATTCGTAATG	CTGCCCATTCTGCTATTCTG
A		GAAGGGAGGTTCTTAGGTTG	GTTTCCTCTTTGATTAACGC
C		CCACATCATCCACGGTTCTA	ACTGTTGACCCAATGCGTCT
A		AACACACCACAGACGTGGGT	AATACCATTAAAAGATGAGA
-	-1	ACTGTGCCCTCCATGGAAAA	TCAGTCAAGATATCCACATG
C		CGCTGCCTTCACATTTCAAG	TACTGAACTAGTTGGTATCA
-	-1	AACAGGACTAGGATGAGTAG	AGCACGTTCCTTATATGTAG
CAAACCT		TCTTCAACGCTGTTATCTTA	TTATCTCTGCCGCAAATTCA
C		ACAATTCTGCTAACATCAAA	GGCCTCTAGGTTCCTTTATT
A		CGAAATGGCTACATTCATCC	TGTTACATCCTCTTTCACAG
A	+1	ATGTCTCTTTGAGCAATAAA	GCCGATAACAAAATCTTTGT
G		TGGTGCGGCCAATGGTTACA	GTATTGGTTTTCTTGGGCAA
G		CCTTTGCAAATAGTCCTCTT	CAACAATAATAATGTCAGAT
-	-1	TTTGATTAACGCTGCCTTCA	ATTTCAAGGTACTGAACTAG
C		TCTTCCACCCATGTCTCTTT	AGCAATAAAGCCGATAACAA
-	-1	GATGAAGATGAAGGAGAAGT	CAGAACGCTGAAGTGAAGAG
G		GACTGTACTACCAATGTCAG	AAATTTTCTGTCTTCGAAGA
A		GCCAATGGTTACATGTATTG	TTTTCTTGGGCAATCACTTT
TT		GGATGAGTAGCAGCACGTTC	TTATATGTAGCTTTCGACAT
A		CATCAAAAAAGTTGTTTTCC	TATCTTAACCTTCTACATTG
T		AATGACCCTAAACTAACACA	TCTACTTCCTACGTTTCTAC
A		ATTCAGATGCAGATTTATTT	GAAGATTGGAGATGTCGACA
-	-1	CTTCAACTAACTCCAGTAAT	CCTTGGTGGTACGAACATCC
G		CAAATATTTACGTTGGTTCC	GTATTTTATTTGGTCTATCA
A		GGCAGCAACAGGACTAGGAT	AGTAGCAGCACGTTCCTTAT
A		GGCATGGATTAGTATTTTTT	GGTAATTATCACAATAATGA
-	-1	TATCCACATGTGTTTTTAGT	AACAAATTTTGGGACCTAAT
A		GGCCAATGGTTACATGTATT	GTTTTCTTGGGCAATCACTT
A		GTACTTGGCGGATAATGCCT	TAGCGGCTTAACTGTGCCCT
T		ACTAGTTGGTATCACTGCTG	TGAAGCTGCAAACCCCAGAA
T		ATTCTGCTATTCTGTATACA	CCGCAGAGTACTGCAATTTG
A		CCCTGTTACATCCTCTTTCA	AGTGTTCTCACAAAGATTCC
A		GTCTCTTTGAGCAATAAAGC	GATAACAAAATCTTTGTCGC
C		CGGCCAATGGTTACATGTAT	GGTTTTCTTGGGCAATCACT
T		ACTAGGATGAGTAGCAGCAC	TTCCTTATATGTAGCTTTCG
A		GGGCAATCACTTTTGCCCTG	AACTTAGTGTAGTTGGCCAA
TTGA		CGGCTTAACTGTGCCCTCCA	AAAAATCAGTCAAGATATCC

AAG	TTTCTAATATACTGTTTTTG	GGTTTGTGGTGCTGGGGTTA
G	TCTCAAATATGCTTCCCAGC	TGCTTTTCTGTAACGTTCAC
G	GTTATTTATCTCAATCTCGC	CATCAGATTTATGCAAGCTT
C	CATTTAGAGCTAAATTAATG	CCGGCTTGGCTTATTATGCG
A	CATTTAGAGCTAAATTAATG	CCGGCTTGGCTTATTATGCG
G	CAAATTTTCTGTCTTCGAAG	GTAAAAAATTGTACTTGGCG
C	TGCTAACATCAAAAGGCCTC	AGGTTCCTTTATTACTTCTT
A	GGCATGGATTAGTATTTTTT	GGTAATTATCACAATAATGA

type of mutation	
complex	1
substitution	2
substitution	3
deletion	4
complex	5
complex	6
substitution	7
substitution	8
deletion	9
substitution	10
complex	11
substitution	12
substitution	13
substitution	14
substitution	15
substitution	16
complex	17
substitution	18
complex	19
substitution	20
substitution	21
substitution	22
substitution	23
substitution	24
substitution	25
substitution	26
substitution	27
substitution	28
substitution	29
substitution	30
substitution	31
substitution	32
substitution	33
complex	34
substitution	35
substitution	36
substitution	37

substitution	1
substitution	2
substitution	3
substitution	4
substitution	5
substitution	6
substitution	7
substitution	8
substitution	9
substitution	10
substitution	11
substitution	12
substitution	13
substitution	14
substitution	15
substitution	16
substitution	17
substitution	18
substitution	19
substitution	20
substitution	21
complex	22
substitution	23
substitution	24
substitution	25
substitution	26
deletion	27
substitution	28
substitution	29
substitution	30
substitution	31
substitution	32
deletion	33
deletion	34
deletion	35
substitution	36
substitution	37
deletion	38
substitution	39
substitution	40
substitution	41
substitution	42
substitution	43

substitution	44
complex	45
substitution	46
substitution	47
substitution	48
substitution	49
substitution	50
deletion	51
deletion	52
substitution	53
substitution	54
substitution	55
substitution	56
deletion	1
substitution	2
deletion	3
complex	4
substitution	5
substitution	6
insertion	7
substitution	8
substitution	9
deletion	10
substitution	11
deletion	12
substitution	13
substitution	14
complex	15
substitution	16
substitution	17
substitution	18
deletion	19
substitution	20
substitution	21
substitution	22
deletion	23
substitution	24
substitution	25
substitution	26
substitution	27
substitution	28
substitution	29
substitution	30
substitution	31
substitution	32
complex	33

complex	34
substitution	35
substitution	36
substitution	37
substitution	38
substitution	39
substitution	40
substitution	41

mutant strain	relevant genotype	spontaneous or induced?	mutated gene	position in Chr. V (distance from telomere)	WT base(s)
LHY1201	WT	Spontaneous	*CAN1*	6217	C
LHY1202	WT	Spontaneous	*CAN1*	6078	G
LHY1203	WT	Spontaneous	*CAN1*	5774	G
LHY1204	WT	Spontaneous	*CAN1*	6577	GC
LHY1205	WT	Spontaneous	*CAN1*	5673	C
LHY1206	WT	Spontaneous	*CAN1*	5865	-
LHY1207	WT	Spontaneous	*CAN1*	5880	C
LHY1208	WT	Spontaneous	*CAN1*	5679	C
LHY1221	WT	Spontaneous	*CAN1*	5844	T
LHY1222	WT	Spontaneous	*CAN1*	5694	C
LHY1223	WT	Spontaneous	*CAN1*	5513	G
LHY1224	WT	Spontaneous	*CAN1*	5989	C
LHY1225	WT	Spontaneous	*CAN1*	5879	C
LHY1226	WT	Spontaneous	*CAN1*	6419	C
LHY1301	WT	Spontaneous	*CAN1*	6732	C
LHY1302	WT	Spontaneous	*CAN1*	5640	C
LHY1303	WT	Spontaneous	*CAN1*	5546	-
LHY1304	WT	Spontaneous	*CAN1*	6717	T
LHY1305	WT	Spontaneous	*CAN1*	5514	C
LHY1306	WT	Spontaneous	*CAN1*	6419	C
LHY1307	WT	Spontaneous	*CAN1*	5505	G
LHY1308	WT	Spontaneous	*CAN1*	5850	-
LHY1401	WT	Spontaneous	*CAN1*	6633	C
LHY1402	WT	Spontaneous	*CAN1*	6492	C
LHY1403	WT	Spontaneous	*CAN1*	5589	C
LHY1404	WT	Spontaneous	*CAN1*	6390	C
LHY1405	WT	Spontaneous	*CAN1*	5751	C
LHY1406	WT	Spontaneous	*CAN1*	6194	G
LHY1407	WT	Spontaneous	*CAN1*	6081	G
LHY1408	WT	Spontaneous	*CAN1*	6318	C

mutant base(s)	deletion/insertion (-/+ number of bases)	5′ flanking sequence	3′ flanking sequence
A		GTTTTCCGTATCTTAACCTT	TACATTGGCTCTCTATTATT
-	-1	AAACGAAGGGAGGTTCTTAG	TTGGGTTTCCTCTTTGATTA
C		GGGCAATCACTTTTGCCCTG	AACTTAGTGTAGTTGGCCAA
AA		GGTGACAAAGTTTTCGAATG	TATTAAATATCACTGGTGTT
A		GGCTACATTCATCCCTGTTA	ATCCTCTTTCACAGTGTTCT
A	+1	TTTTTGGGTAATTATCACAA	TAATGAACTTGTTCCCTGTC
G		CACAATAATGAACTTGTTCC	TGTCAAATATTACGGTGAAT
A		ATTCATCCCTGTTACATCCT	TTTCACAGTGTTCTCACAAA
A		CCACTGGCGGCATGGATTAG	ATTTTTTGGGTAATTATCAC
A		ATCCTCTTTCACAGTGTTCT	ACAAAGATTCCTTTCTCCAG
C		AAAGACATATTGGTATGATT	CCCTTGGTGGTACTATTGGT
-	-1	GTTTGTGGTGCTGGGGTTAC	GGCCCAGTTGGATTCCGTTA
G		TCACAATAATGAACTTGTTC	CTGTCAAATATTACGGTGAA
-	-1	TCAAATATTTACGTTGGTTC	CGTATTTTATTTGGTCTATC
A		CGGCTTGGCTTATTATGCGG	CACATTTATGACGATCATTA
A		GGCATATTCTGTCACGCAGT	CTTGGGCGAAATGGCTACAT
AA		CTATTGGTACAGGTCTTTTC	ATTGGTTTATCCACACCTCT
A		AGCTAAATTAATGCCCGGCT	GGCTTATTATGCGGCCACAT
A		AAGACATATTGGTATGATTG	CCTTGGTGGTACTATTGGTA
A		CAAATATTTACGTTGGTTCC	GTATTTTATTTGGTCTATCA
T		GCTTAAGCAAAGACATATTG	TATGATTGCCCTTGGTGGTA
T	+1	ACTGGCGGCATGGATTAGTA	GGGTAATTATCACAATAATG
G		ATGGTTATTTATCTCAATCT	GCACATCAGATTTATGCAAG
A		GACCACCAAAGGTGGTGTTC	ATACATTGCAGTTTTCGTTA
T		CAACGCCGGCCCAGTGGGCG	TCTTATATCATATTTATTTA
T		CTTAACAACCATTATCTCTG	CGCAAATTCAAATATTTACG
T		TGGTTACATGTATTGGTTTT	TTGGGCAATCACTTTTGCCC
T		TTCCAAGAGCCATCAAAAAA	TTGTTTTCCGTATCTTAACC
A		CGAAGGGAGGTTCTTAGGTT	GGTTTCCTCTTTGATTAACG
A		TACTTCTCCCTTTATTATTG	TATTGAGAACTCTGGTACAA

type of mutation
substitution
deletion
substitution
complex
substitution
insertion
substitution
substitution
substitution
substitution
substitution
deletion
substitution
deletion
substitution
substitution
substitution
substitution
substitution
substitution
substitution
insertion
substitution
substitution
substitution
substitution
substitution
substitution
substitution
substitution

mutant strain	relevant genotype	spontaneous or induced?	mutated gene	position in Chr. V (distance from telomere)	WT base(s)
LHY2001	*rev3 Δ*	Spontaneous	*CAN1*	6188	A
LHY2002	*rev3 Δ*	Spontaneous	*CAN1*	5444	[illegible]TGAAGGAGAA
LHY2003	*rev3 Δ*	Spontaneous	*CAN1*	5755	G
LHY2004	*rev3 Δ*	Spontaneous	*CAN1*	5882	G
LHY2005	*rev3 Δ*	Spontaneous	*CAN1*	5522	G
LHY2006	*rev3 Δ*	Spontaneous	*CAN1*	5984	G
LHY2007	*rev3 Δ*	Spontaneous	*CAN1*	6331	T
LHY2008	*rev3 Δ*	Spontaneous	*CAN1*	6416	T
LHY2010	*rev3 Δ*	Spontaneous	*CAN1*	6423	T
LHY2011	*rev3 Δ*	Spontaneous	*CAN1*	6183	C
LHY2012	*rev3 Δ*	Spontaneous	*CAN1*	5964	-
	rev3 Δ	Spontaneous	*CAN1*	5260	-
LHY2013	*rev3 Δ*	Spontaneous	*CAN1*	6183	C
LHY2014	*rev3 Δ*	Spontaneous	*CAN1*	6446	A
LHY2015	*rev3 Δ*	Spontaneous	*CAN1*	6579	T
LHY2016	*rev3 Δ*	Spontaneous	*CAN1*	5850	-
LHY2018	*rev3 Δ*	Spontaneous	*CAN1*	6188	A
LHY2019	*rev3 Δ*	Spontaneous	*CAN1*	6605	T
LHY2020	*rev3 Δ*	Spontaneous	*CAN1*	6610	T
LHY2021	*rev3 Δ*	Spontaneous	*CAN1*	5880	C
LHY2022	*rev3 Δ*	Spontaneous	*CAN1*	5520	G
LHY2023	*rev3 Δ*	Spontaneous	*CAN1*	5522	G
LHY2024	*rev3 Δ*	Spontaneous	*CAN1*	6390	C
LHY2026	*rev3 Δ*	5mM H_2O_2	*CAN1*	5379	T
LHY2027	*rev3 Δ*	5mM H_2O_2	*CAN1*	5523	G
LHY2028	*rev3 Δ*	5mM H_2O_2	*CAN1*	5537	G
LHY2029	*rev3 Δ*	5mM H_2O_2	*CAN1*	5888	T
LHY2030	*rev3 Δ*	5mM H_2O_2	*CAN1*	5774	G
LHY2031	*rev3 Δ*	5mM H_2O_2	*CAN1*	6234	T
LHY2032	*rev3 Δ*	5mM H_2O_2	*CAN1*	5746	G
LHY2033	*rev3 Δ*	5mM H_2O_2	*CAN1*	5894	G
LHY2034	*rev3 Δ*	5mM H_2O_2	*CAN1*	5774	G
LHY2035	*rev3 Δ*	5mM H_2O_2	*CAN1*	6486	G
LHY2036	*rev3 Δ*	5mM H_2O_2	*CAN1*	5588	G
LHY2037	*rev3 Δ*	5mM H_2O_2	*CAN1*	5576	G
LHY2038	*rev3 Δ*	5mM H_2O_2	*CAN1*	5733	G

LHY2039	*rev3 Δ*	5mM H_2O_2	*CAN1*	6762	A
LHY2040	*rev3 Δ*	5mM H_2O_2	*CAN1*	6594	G
LHY2041	*rev3 Δ*	5mM H_2O_2	*CAN1*	6147	G
LHY2042	*rev3 Δ*	5mM H_2O_2	*CAN1*	5522	G
LHY2043	*rev3 Δ*	5mM H_2O_2	*CAN1*	5809	G
LHY2044	*rev3 Δ*	5mM H_2O_2	*CAN1*	6188	A
LHY2045	*rev3 Δ*	5mM H_2O_2	*CAN1*	5807	T
LHY2046	*rev3 Δ*	5mM H_2O_2	*CAN1*	6062	G
LHY2047	*rev3 Δ*	5mM H_2O_2	*CAN1*	6420	G
LHY2048	*rev3 Δ*	5mM H_2O_2	*CAN1*	6119	G

mutant base(s)	deletion/insertion (-/+ number of bases)	5′ flanking sequence	3′ flanking sequence
-	-1	AATCCGTTCCAAGAGCCATC	GTTGTTTTCCGTATCTTAAC
[illegible]GTACA	-20	TGGAGGATGGCATAGGTGAT	GAACGCTGAAGTGAAGAGAG
A		TACATGTATTGGTTTTCTTG	GCAATCACTTTTGCCCTGGA
A		CAATAATGAACTTGTTCCCT	TCAAATATTACGGTGAATTC
A		TTGGTATGATTGCCCTTGGT	GTACTATTGGTACAGGTCTT
-	-1	TTTGTATGGTTTGTGGTGCT	TTACCGGCCCAGTTGGATTC
-	-1	ATTATTGCTATTGAGAACTC	GGTACAAAGGTTTTGCCACA
-	-1	AATTCAAATATTTACGTTGG	TCCCGTATTTTATTTGGTCT
-	-1	TATTTACGTTGGTTCCCGTA	ATTTGGTCTATCAAAGAACA
T		CAGAAAATCCGTTCCAAGAG	CATCAAAAAAGTTGTTTTCC
T	+1	ATCGGGTTTCTAATATACTG	GTATGGTTTGTGGTGCTGGG
G	+1	GAAGACGCCGACATAGAGGA	GAAGCATATGTACAATGAGC
T		CAGAAAATCCGTTCCAAGAG	CATCAAAAAAGTTGTTTTCC
-	-1	ATTTGGTCTATCAAAGAACA	AGTTGGCTCCTAAATTCCTG
C		TGACAAAGTTTTCGAATGGC	ATTAAATATCACTGGTGTTG
T	+1	ACTGGCGGCATGGATTAGTA	GGGTAATTATCACAATAATG
-	-1	AATCCGTTCCAAGAGCCATC	GTTGTTTTCCGTATCTTAAC
-	-1	ATATCACTGGTGTTGCAGGC	GCATGGTTATTTATCTCAAT
-	-1	ATATCACTGGTGTTGCAGGC	GCATGGTTATTTATCTCAAT
T		CACAATAATGAACTTGTTCC	TGTCAAATATTACGGTGAAT
A		TATTGGTATGATTGCCCTTG	TGGTACTATTGGTACAGGTC
T		TTGGTATGATTGCCCTTGGT	GTACTATTGGTACAGGTCTT
T		CTTAACAACCATTATCTCTG	CGCAAATTCAAATATTTACG
A		AAGTAAAGAATTGCATCCAT	GCGCTCTTTCCCGACGAGAG
C		TGGTATGATTGCCCTTGGTG	TACTATTGGTACAGGTCTTT
A		TTGGTGGTACTATTGGTACA	GTCTTTTCATTGGTTTATCC
A		TGAACTTGTTCCCTGTCAAA	ATTACGGTGAATTCGAGTTC
A		GGGCAATCACTTTTGCCCTG	AACTTAGTGTAGTTGGCCAA
A		CTTCTACATTGGCTCTCTAT	ATTCATTGGAC-TTTTAGTTC
A		GCCAATGGTTACATGTATTG	TTTTCTTGGGCAATCACTTT
T		TGTTCCCTGTCAAATATTAC	GTGAATTCGAGTTCTGGGTC
A		GGGCAATCACTTTTGCCCTG	AACTTAGTGTAGTTGGCCAA
A		GTCAAGGACCACCAAAGGTG	TGTTCCATACATTGCAGTTT
C		CCAACGCCGGCCCAGTGGGC	CTCTTATATCATATTTATTT
A		CCACACCTCTGACCAACGCC	GCCCAGTGGGCGCTCTTATA
A		AGCATTTGGTGCGGCCAATG	TTACATGTATTGGTTTTCTT

G		GACGATCATTATCATTATTC	AGGTTTCACGGCTTTTGCAC
A		ATGGCTATTAAATATCACTG	TGTTGCAGGCTTTTTTGCAT
C		ACTAGTTGGTATCACTGCTG	TGAAGCTGCAAACCCCAGAA
A		TTGGTATGATTGCCCTTGGT	GTACTATTGGTACAGGTCTT
A		GGCCAAGTCATTCAATTTTG	ACGTACA-AAGTTCCACTGGC
-	-1	AATCCGTTCCAAGAGCCATC	GTTGTTTTCCGTATCTTAAC
-	-1	TAGTTGGCCAAGTCATTCAA	GGACGTACAAAGTTCCACTG
T		TAATATCTAAGGATAAAAAC	AAGGGAGGTTCTTAGGTTGG
A		AAATATTTACGTTGGTTCCC	TATTTTATTTGGTCTATCAA
A		ACGCTGCCTTCACATTTCAA	GTACTGAACTAGTTGGTATC

type of mutation
deletion
deletion
substitution
substitution
substitution
deletion
deletion
deletion
deletion
substitution
insertion
insertion
substitution
deletion
substitution
insertion
deletion
deletion
deletion
substitution
substitution
substitution
substitution
substitution
substitution
substitution
substitution
substitution
substitution
substitution
substitution
substitution
substitution
substitution
substitution
substitution

substitution
substitution
substitution
substitution
substitution
deletion
deletion
substitution
substitution
substitution

mutant strain	relevant genotype	spontaneous or induced?	mutated gene	position in Chr. V (distance from telomere)	WT base(s)
LHY1001	*rev3Δ cta1Δ*	Spontaneous	*CAN1*	6390	C
LHY1002	*rev3Δ cta1Δ*	Spontaneous	*CAN1*	6602	G
LHY1003	*rev3Δ cta1Δ*	Spontaneous	*CAN1*	6147	G
LHY1004	*rev3Δ cta1Δ*	Spontaneous	*CAN1*	6534	C
LHY1005	*rev3Δ cta1Δ*	Spontaneous	*CAN1*	5880	C
LHY1006	*rev3Δ cta1Δ*	Spontaneous	*CAN1*	5912	G
LHY1007	*rev3Δ cta1Δ*	Spontaneous	*CAN1*	6205-6352	TAACACAATCT[illegible]
LHY1008	*rev3Δ cta1Δ*	Spontaneous	*CAN1*	5520	G
LHY1011	*rev3Δ cta1Δ*	Spontaneous	*CAN1*	6417	C
LHY1012	*rev3Δ cta1Δ*	Spontaneous	*CAN1*	6203	C
LHY1013	*rev3Δ cta1Δ*	Spontaneous	*CAN1*	6419	C
LHY1014	*rev3Δ cta1Δ*	Spontaneous	*CAN1*	5950	-
LHY1015	*rev3Δ cta1Δ*	Spontaneous	*CAN1*	6065	G
LHY1016	*rev3Δ cta1Δ*	Spontaneous	*CAN1*	6161	C
LHY1017	*rev3Δ cta1Δ*	Spontaneous	*CAN1*	6248	T
LHY1018	*rev3Δ cta1Δ*	Spontaneous	*CAN1*	5499	A
LHY1019	*rev3Δ cta1Δ*	Spontaneous	*CAN1*	6594	G
LHY1020	*rev3Δ cta1Δ*	Spontaneous	*CAN1*	5531	GG
LHY1021	*rev3Δ cta1Δ*	Spontaneous	*CAN1*	6496	C
LHY1022	*rev3Δ cta1Δ*	Spontaneous	*CAN1*	6188	A
LHY1023	*rev3Δ cta1Δ*	Spontaneous	*CAN1*	5751	C
LHY1024	*rev3Δ cta1Δ*	Spontaneous	*CAN1*	5589	C
LHY1101	*rev3Δ cta1Δ*	5mM H_2O_2	*CAN1*	6594	G
LHY1102	*rev3Δ cta1Δ*	5mM H_2O_2	*CAN1*	6013	G
LHY1103	*rev3Δ cta1Δ*	5mM H_2O_2	*CAN1*	5951-6294	
LHY1104	*rev3Δ cta1Δ*	5mM H_2O_2	*CAN1*	5733	G
LHY1105	*rev3Δ cta1Δ*	5mM H_2O_2	*CAN1*	5645	G
LHY1106	*rev3Δ cta1Δ*	5mM H_2O_2	*CAN1*	5956	C
LHY1107	*rev3Δ cta1Δ*	5mM H_2O_2	*CAN1*	5911	G
LHY1108	*rev3Δ cta1Δ*	5mM H_2O_2	*CAN1*	5621	G
LHY1109	*rev3Δ cta1Δ*	5mM H_2O_2	*CAN1*	5808	G
LHY1110	*rev3Δ cta1Δ*	5mM H_2O_2	*CAN1*	5733	G
LHY1111	*rev3Δ cta1Δ*	5mM H_2O_2	*CAN1*	6149	G
LHY1112	*rev3Δ cta1Δ*	5mM H_2O_2	*CAN1*	6010	T
LHY1113	*rev3Δ cta1Δ*	5mM H_2O_2	*CAN1*	5640	C
LHY1114	*rev3Δ cta1Δ*	5mM H_2O_2	*CAN1*	5809	G
LHY1115	*rev3Δ cta1Δ*	5mM H_2O_2	*CAN1*	5531	G
LHY1116	*rev3Δ cta1Δ*	5mM H_2O_2	*CAN1*	6082	G

LHY1117	*rev3Δ cta1Δ*	5mM H_2O_2	*CAN1*	5228	A
LHY1118	*rev3Δ cta1Δ*	5mM H_2O_2	*CAN1*	5381	C
LHY1119	*rev3Δ cta1Δ*	5mM H_2O_2	*CAN1*	5531	G
LHY1120	*rev3Δ cta1Δ*	5mM H_2O_2	*CAN1*	6486	G
LHY1121	*rev3Δ cta1Δ*	5mM H_2O_2	*CAN1*	5721	G
LHY1122	*rev3Δ cta1Δ*	5mM H_2O_2	*CAN1*	6594	G
LHY1123	*rev3Δ cta1Δ*	5mM H_2O_2	*CAN1*	5531	G
LHY1124	*rev3Δ cta1Δ*	5mM H_2O_2	*CAN1*	5648	G

mutant base(s)	deletion/insertion (-/+ number of bases)	5′ flanking sequence	3′ flanking sequence
T		CTTAACAACCATTATCTCTG	CGCAAATTCAAATATTTACG
A		TAAATATCACTGGTGTTGCA	GCTTTTTTGCATGGTTATTT
A		ACTAGTTGGTATCACTGCTG	TGAAGCTGCAAACCCCAGAA
A		TGCTGCATTTGGCGCTTTGG	TTACATGGAGACATCTACTG
-	-1	CACAATAATGAACTTGTTCC	TGTCAAATATTACGGTGAA
-	-1	ACGGTGAATTCGAGTTCTGG	TCGCTTCCATCAAAGTTTTA
ACTTCCTACGT	-147	ATCAAAAAAGTTGTTTTCCG	TATCTTCAACGCTGTTATCT
A		TATTGGTATGATTGCCCTTG	TGGTACTATTGGTACAGGTC
-	-1	CAAATATTTACGTTGGTTCC	GTATTTTATTTGGTCTATCA
T		CCATCAAAAAAGTTGTTTTC	GTATCTTAACCTTCTACATT
A		CAAATATTTACGTTGGTTCC	GTATTTTATTTGGTCTATCA
T	+1	GGCATGGATTAGTATTTTTT	GGGTAATTATCACAATAATG
-	-1	TATCTAAGGATAAAAACGAA	GGAGGTTCTTAGGTTGGGTT
A		CTGCTGGTGAAGCTGCAAAC	CCAGAAAATCCGTTCCAAGA
-	-1	CTCTCTATTATTCATTGGAC	TTAGTTCCATACAATGACCC
G		GAGAGAGCTTAAGCAAAGAC	TATTGGTATGATTGCCCTTG
A		ATGGCTATTAAATATCACTG	TGTTGCAGGCTTTTTTGCAT
AA		TTGCCCTTGGTGGTACTATT	TACAGGTCTTTTCATTGGTT
A		ACCAAAGGTGGTGTTCCATA	ATTGCAGTTTTCGTTACTGC
-	-1	AATCCGTTCCAAGAGCCATC	AAAAAGTTGTTTTCCGTATC
A		TGGTTACATGTATTGGTTTT	TTGGGCAATCACTTTTGCCC
A		CAACGCCGGCCCAGTGGGCG	TCTTATATCATATTTATTTA
A		ATGGCTATTAAATATCACTG	TGTTGCAGGCTTTTTTGCAT
A		CCAGTTGGATTCCGTTATTG	AGAAACCCAGGTGCCTGGGG
	-343	TAGCCATTATCGGGTTTCTA	TTCTACTTCTCCCTTTATTA
A		AGCATTTGGTGCGGCCAATG	TTACATGTATTGGTTTTCTT
A		ATTCTGTCACGCAGTCCTTG	GCGAAATGGCTACATTCATC
A		ATTATCGGGTTTCTAATATA	TGTTTTTGTATGGTTTGTGG
A		TACGGTGAATTCGAGTTCTG	GTCGCTTCCATCAAAGTTTT
C		ATTTATTTATGGGTTCTTTG	CATATTCTGTCACGCAGTCC
A		TGGCCAAGTCATTCAATTTT	GACGTACAAAGTTCCACTGG
A		AGCATTTGGTGCGGCCAATG	TTACATGTATTGGTTTTCTT
A		TAGTTGGTATCACTGCTGGT	AAGCTGCAAACCCCAGAAAA
A		GGCCCAGTTGGATTCCGTTA	TGGAGAAACCCAGGTGCCTG
A		GGCATATTCTGTCACGCAGT	CTTGGGCGAAATGGCTACAT
A		GGCCAAGTCATTCAATTTTG	ACGTACAAAGTTCCACTGGC
T		TTGCCCTTGGTGGTACTATT	GTACAGGTCTTTTCATTGGT
A		GAAGGGAGGTTCTTAGGTTG	GTTTCCTCTTTGATTAACGC

T		AAAAAAAAGGCATAGCAATG	CAAATTCAAAAGAAGACGCC
-	-1	GTAAAGAATTGCATCCATTG	GCTCTTTCCCGACGAGAGTA
T		TTGCCCTTGGTGGTACTATT	GTACAGGTCTTTTCATTGGT
A		GTCAAGGACCACCAAAGGTG	TGTTCCATACATTGCAGTTT
A		ATTCCTTTCTCCAGCATTTG	TGCGGCCAATGGTTACATGT
A		ATGGCTATTAAATATCACTG	TGTTGCAGGCTTTTTTGCAT
C		TTGCCCTTGGTGGTACTATT	GTACAGGTCTTTTCATTGGT
A		CTGTCACGCAGTCCTTGGGC	AAATGGCTACATTCATCCCT

type of mutation
substitution
substitution
substitution
substitution
deletion
deletion
deletion
substitution
deletion
substitution
substitution
insertion
deletion
substitution
deletion
substitution
substitution
complex
substitution
deletion
substitution
substitution
substitution
substitution
deletion
substitution
substitution
substitution
substitution
substitution
substitution
substitution
substitution
substitution
substitution
substitution
substitution
substitution

substitution
deletion
substitution
substitution
substitution
substitution
substitution
substitution

mutant strain	relevant genotype	spontaneous or induced?	mutated gene	position in Chr. V (distance from telomere)	WT base(s)
LHY701	*rev3 Δ sod1 Δ*	Spontaneous	*CAN1*	5809	G
LHY702	*rev3 Δ sod1 Δ*	Spontaneous	*CAN1*	6486	G
LHY703	*rev3 Δ sod1 Δ*	Spontaneous	*CAN1*	6419	C
LHY704	*rev3 Δ sod1 Δ*	Spontaneous	*CAN1*	6762	A
LHY706	*rev3 Δ sod1 Δ*	Spontaneous	*CAN1*	6572	G
LHY707	*rev3 Δ sod1 Δ*	Spontaneous	*CAN1*	6293	G
LHY708	*rev3 Δ sod1 Δ*	Spontaneous	*CAN1*	6409	C
LHY709	*rev3 Δ sod1 Δ*	Spontaneous	*CAN1*	6534	C
LHY710	*rev3 Δ sod1 Δ*	Spontaneous	*CAN1*	6165	G
LHY711	*rev3 Δ sod1 Δ*	Spontaneous	*CAN1*	5963	T
LHY712	*rev3 Δ sod1 Δ*	Spontaneous	*CAN1*	6605	-
LHY713	*rev3 Δ sod1 Δ*	Spontaneous	*CAN1*	6144	C
LHY714	*rev3 Δ sod1 Δ*	Spontaneous	*CAN1*	6438	C
LHY715	*rev3 Δ sod1 Δ*	Spontaneous	*CAN1*	6188	A
LHY716	*rev3 Δ sod1 Δ*	Spontaneous	*CAN1*	6521	T
LHY717	*rev3 Δ sod1 Δ*	Spontaneous	*CAN1*	5903	G
LHY718	*rev3 Δ sod1 Δ*	Spontaneous	*CAN1*	5844	-
	rev3 Δ sod1 Δ	Spontaneous	*CAN1*	6841	T
LHY719	*rev3 Δ sod1 Δ*	Spontaneous	*CAN1*	5911	G
LHY720	*rev3 Δ sod1 Δ*	Spontaneous	*CAN1*	5513	G
LHY721	*rev3 Δ sod1 Δ*	Spontaneous	*CAN1*	6305	C
LHY722	*rev3 Δ sod1 Δ*	Spontaneous	*CAN1*	5752	TT
LHY723	*rev3 Δ sod1 Δ*	Spontaneous	*CAN1*	6134	G
LHY724	*rev3 Δ sod1 Δ*	Spontaneous	*CAN1*	6125	G
LHY301	*rev3 Δ sod1 Δ*	150uM paraquat	*CAN1*	5815	C
LHY302	*rev3 Δ sod1 Δ*	150uM paraquat	*CAN1*	5520	G
LHY303	*rev3 Δ sod1 Δ*	150uM paraquat	*CAN1*	6161	C
LHY304	*rev3 Δ sod1 Δ*	150uM paraquat	*CAN1*	5746	G
LHY305	*rev3 Δ sod1 Δ*	150uM paraquat	*CAN1*	6084	G
LHY306	*rev3 Δ sod1 Δ*	150uM paraquat	*CAN1*	5537	G
LHY307	*rev3 Δ sod1 Δ*	150uM paraquat	*CAN1*	6409	C
LHY309	*rev3 Δ sod1 Δ*	150uM paraquat	*CAN1*	6031	G
	rev3 Δ sod1 Δ	150uM paraquat	*CAN2*	5846	TTTTGG
LHY310	*rev3 Δ sod1 Δ*	150uM paraquat	*CAN1*	6846	G
LHY311	*rev3 Δ sod1 Δ*	150uM paraquat	*CAN1*	5808	G
LHY312	*rev3 Δ sod1 Δ*	150uM paraquat	*CAN1*	6846	G
LHY313	*rev3 Δ sod1 Δ*	150uM paraquat	*CAN1*	5685	C
LHY314	*rev3 Δ sod1 Δ*	150uM paraquat	*CAN1*	5532	G

LHY315	*rev3Δ sod1Δ*	150uM paraquat	*CAN1*	5745	G
LHY316	*rev3Δ sod1Δ*	150uM paraquat	*CAN1*	5755	G
LHY317	*rev3Δ sod1Δ*	150uM paraquat	*CAN1*	6417	C
LHY319	*rev3Δ sod1Δ*	150uM paraquat	*CAN1*	6068	A
LHY320	*rev3Δ sod1Δ*	150uM paraquat	*CAN1*	5745	G
LHY321	*rev3Δ sod1Δ*	150uM paraquat	*CAN1*	6117	A
LHY322	*rev3Δ sod1Δ*	150uM paraquat	*CAN1*	6468	C
LHY323	*rev3Δ sod1Δ*	150uM paraquat	*CAN1*	5850	-
LHY324	*rev3Δ sod1Δ*	150uM paraquat	*CAN1*	6204	G

mutant base(s)	deletion/insertion (-/+ number of bases)	5′ flanking sequence	3′ flanking sequence
A		GGCCAAGTCATTCAATTTTG	ACGTACAAAGTTCCACTGGC
A		GTCAAGGACCACCAAAGGTG	TGTTCCATACATTGCAGTTT
T		CAAATATTTACGTTGGTTCC	GTATTTTATTTGGTCTATCA
G		GACGATCATTATCATTATTC	AGGTTTCACGGCTTTTGCAC
T		CTGGTGGTGACAAAGTTTTC	AATGGCTATTAAATATCACT
-	-1	TAACACAATCTACTTCCTAC	TTTCTACTTCTCCCTTTATT
A		GCCGCAAATTCAAATATTTA	GTTGGTTCCCGTATTTTATT
A		TGCTGCATTTGGCGCTTTGG	TTACATGGAGACATCTACTG
-	-1	TGGTGAAGCTGCAAACCCCA	AAAATCCGTTCCAAGAGCCA
-	-1	ATCGGGTTTCTAATATACTG	TTTTGTATGGTTTGTGGTGC
T	+1	ATATCACTGGTGTTGCAGGC	TTTTTTGCATGGTTATTTAT
A		TGAACTAGTTGGTATCACTG	TGGTGAAGCTGCAAACCCCA
A		CCGTATTTTATTTGGTCTAT	AAAGAACAAGTTGGCTCCTA
-	-1	AATCCGTTCCAAGAGCCATC	AAAAAGTTGTTTTCCGTATC
-	-1	CAGTTTTCGTTACTGCTGCA	TTGGCGCTTTGGCTTACATG
A		TCAAATATTACGGTGAATTC	AGTTCTGGGTCGCTTCCATC
T	+1	GGCATGGATTAGTATTTTTT	GGGTAATTATCACAATAATG
G		TCTGTTTTCCTGTTCTTAGC	GTTTGGATCTTATTTCAATG
A		TACGGTGAATTCGAGTTCTG	GTCGCTTCCATCAAAGTTTT
C		AAAGACATATTGGTATGATT	CCCTTGGTGGTACTATTGGT
-	-1	CTTCCTACGTTTCTACTTCT	CCTTTATTATTGCTATTGAG
CA		GGTTACATGTATTGGTTTTC	GGGCAATCACTTTTGCCCTG
C		TTCAAGGTACTGAACTAGTT	GTATCACTGCTGGTGAAGCT
A		CCTTCACATTTCAAGGTACT	AACTAGTTGGTATCACTGCT
A		GTCATTCAATTTTGGACGTA	AAAGTTCCACTGGCGGCATG
A		TATTGGTATGATTGCCCTTG	TGGTACTATTGGTACAGGTC
A		CTGCTGGTGAAGCTGCAAAC	CCAGAAAATCCGTTCCAAGA
A		GCCAATGGTTACATGTATTG	TTTTCTTGGGCAATCACTTT
A		CAATAATGAACTTGTTCCCT	TCAAATATTACGGTGAATTC
A		TTGGTGGTACTATTGGTACA	GTCTTTTCATTGGTTTATCC
A		GCCGCAAATTCAAATATTTA	GTTGGTTCCCGTATTTTATT
A		TGGAGAAACCCAGGTGCCTG	GGTCCAGGTATAATATCTAA
CTTTGT		TGGCGGCATGGATTAGTATT	GTAATTATCACAATAATGAA
A		TTTCCTGTTCTTAGCTGTTT	GATCTTATTTCAATGCATAT
A		TGGCCAAGTCATTCAATTTT	GACGTACAAAGTTCCACTGG
A		TTTCCTGTTCTTAGCTGTTT	GATCTTATTTCAATGCATAT
A		CCCTGTTACATCCTCTTTCA	AGTGTTCTCACAAAGATTCCT
A		TGCCCTTGGTGGTACTATTG	TACAGGTCTTTTCATTGGTT

A		GGCCAATGGTTACATGTATT	GTTTTCTTGGGCAATCACTT
T		TACATGTATTGGTTTTCTTG	GCAATCACTTTTGCCCTGGA
G		TTCAAATATTTACGTTGGTT	CCGTATTTTATTTGGTCTAT
-	-1	CTAAGGATAAAAACGAAGGG	GGTTCTTAGGTTGGGTTTCC
A		GGCCAATGGTTACATGTATT	GTTTTCTTGGGCAATCACTT
G		TAACGCTGCCTTCACATTTC	AGGTACTGAACTAGTTGGTA
A		GTTGGCTCCTAAATTCCTGT	AAGGACCACCAAAGGTGGTG
T	+1	ACTGGCGGCATGGATTAGTA	GGGTAATTATCACAATAATG
A		CATCAAAAAAGTTGTTTTCC	TATCTTAACCTTCTACATTG

type of mutation
substitution
substitution
substitution
substitution
substitution
deletion
substitution
substitution
deletion
deletion
insertion
substitution
substitution
deletion
deletion
substitution
insertion
substitution
substitution
substitution
deletion
complex
substitution
substitution
substitution
substitution
substitution
substitution
substitution
substitution
substitution
substitution
complex
substitution
substitution
substitution
substitution
substitution

substitution
substitution
substitution
deletion
substitution
substitution
substitution
insertion
substitution

mutant strain	relevant genotype	spontaneous or induced?	mutated gene	position in Chr. V (distance from telomere)	WT base(s)
LH2002	*ung1 Δ*	5mM H_2O_2	*CAN1*	5903	G
LH2003	*ung1 Δ*	5mM H_2O_2	*CAN1*	6162	C
LH2004	*ung1 Δ*	5mM H_2O_2	*CAN1*	5532	G
LH2005	*ung1 Δ*	5mM H_2O_2	*CAN1*	5905	G
LH2006	*ung1 Δ*	5mM H_2O_2	*CAN1*	6605	T
LH2007	*ung1 Δ*	5mM H_2O_2	*CAN1*	5585	G
LH2008	*ung1 Δ*	5mM H_2O_2	*CAN1*	5911	G
LH2010	*ung1 Δ*	5mM H_2O_2	*CAN1*	6594	G
LH2011	*ung1 Δ*	5mM H_2O_2	*CAN1*	5585	G
LH2012	*ung1 Δ*	5mM H_2O_2	*CAN1*	6119	G
LH2031	*ung1 Δ*	5mM H_2O_2	*URA3*	4192	G
	ung1 Δ	5mM H_2O_2	*CAN1*	5942	G
LH2032	*ung1 Δ*	5mM H_2O_2	*URA3*	3461	T
	ung1 Δ	5mM H_2O_2	*CAN1*	6174	T
LH2033	*ung1 Δ*	5mM H_2O_2	*URA3*	4207	T
	ung1 Δ	5mM H_2O_2	*CAN1*	6120	G
LH2034	*ung1 Δ*	5mM H_2O_2	*URA3*	4070	T
	ung1 Δ	5mM H_2O_2	*CAN1*	6012	G
LH2035	*ung1 Δ*	5mM H_2O_2	*URA3*	3976	T
	ung1 Δ	5mM H_2O_2	*CAN1*	6132	T
LH2038	*ung1 Δ*	5mM H_2O_2	*URA3*	4180	A
	ung1 Δ	5mM H_2O_2	*CAN1*	5532	G
LH2039	*ung1 Δ*	5mM H_2O_2	*URA3*	3856	A
	ung1 Δ	5mM H_2O_2	*CAN1*	5654	G
LH2041	*ung1 Δ*	5mM H_2O_2	*URA3*	4062	A
	ung1 Δ	5mM H_2O_2	*CAN1*	5538	G
	ung1 Δ	5mM H_2O_2	*CAN1*	6166-6170	AAAT
LH2043	*ung1 Δ*	5mM H_2O_2	*URA3*	4123	G
	ung1 Δ	5mM H_2O_2	*CAN1*	5485	GC
LH2044	*ung1 Δ*	5mM H_2O_2	*URA3*	4220	G
	ung1 Δ	5mM H_2O_2	*CAN1*	6292	C
LH2046	*ung1 Δ*	5mM H_2O_2	*URA3*	3985	-
	ung1 Δ	5mM H_2O_2	*CAN1*	5603	-TT
LH2047	*ung1 Δ*	5mM H_2O_2	*URA3*	3541	C

	ung1 Δ	5mM H_2O_2	*CAN1*	6030	G
LH2050	*ung1 Δ*	5mM H_2O_2	*URA3*	4039-4048	GAAAAATCAG-
	ung1 Δ	5mM H_2O_2	*CAN1*	5880	C
LH2051	*ung1 Δ*	5mM H_2O_2	*URA3*	4200	A
	ung1 Δ	5mM H_2O_2	*CAN1*	6510	T
LH2052	*ung1 Δ*	5mM H_2O_2	*URA3*	3856	A
	ung1 Δ	5mM H_2O_2	*CAN1*	6033	G
	ung1 Δ	5mM H_2O_2	*CAN1*	6195-6201	TTGTTTT
LH2056	*ung1 Δ*	5mM H_2O_2	*URA3*	3812	T
	ung1 Δ	5mM H_2O_2	*URA3*	3872	C
	ung1 Δ	5mM H_2O_2	*CAN1*	5903	G

mutant base(s)	deletion/insertion (-/+ number of bases)	5′ flanking sequence	3′ flanking sequence
A		GTCAAATATTACGGTGAATTC	AGTTCTGGGTCGCTTCCATC
T		TGCTGGTGAAGCTGCAAACC	CAGAAAATCCGTTCCAAGAG
T		TGCCCTTGGTGGTACTATTG	TACAGGTCTTTTCATTGGTT
C		AAATATTACGGTGAATTCGA	TTCTGGGTCGCTTCCATCAA
-	-1	ACTGGTGTTGCAGGCTTTTT	GCATGGTTATTTATCTCAAT
C		TGACCAACGCCGGCCCAGTG	GCGCTCTTATATCATATTTA
A		TACGGTGAATTCGAGTTCTG	GTCGCTTCCATCAAAGTTTT
A		ATGGCTATTAAATATCACTG	TGTTGCAGGCTTTTTTGCAT
C		TGACCAACGCCGGCCCAGTG	GCGCTCTTATATCATATTTA
T		ACGCTGCCTTCACATTTCAA	GTACTGAACTAGTTGGTATC
T		TGATATTAAATAGCTTGGCA	CAACAGGACTAGGATGAGTA
A		TCAAAGTTTTAGCCATTATC	GGTTTCTAATATACTGTTTT
-	-1	TAGTTTTGCTGGCCGCATCT	CTCAAATATGCTTCCCAGCC
A		TGCAAACCCCAGAAAATCCG	TCCAAGAGCCATCAAAAAAG
A		TGGCAGCAACAGGACTAGGA	GAGTAGCAGCACGTTCCTTA
A		CGCTGCCTTCACATTTCAAG	TACTGAACTAGTTGGTATCA
A		CAAGATATCCACATGTGTTT	TAGTAAACAAATTTTGGGAC
A		CCCAGTTGGATTCCGTTATT	GAGAAACCCAGGTGCCTGGG
A		TGTCAGCAAATTTTCTGTCT	CGAAGAGTAAAAAATTGTAC
A		ATTTCAAGGTACTGAACTAG	TGGTATCACTGCTGGTGAAG
G		GCTTTTCGTGCATGATATTA	ATAGCTTGGCAGCAACAGGA
T		TGCCCTTGGTGGTACTATTG	TACAGGTCTTTTCATTGGTT
T		CCGCCTGCTTCAAACCGCTA	CAATACCTGGACCCACCACA
C		CGCAGTCCTTGGGCGAAATG	CTACATTCATCCCTGTTACA
C		AAATCAGTCAAGATATCCAC	TGTGTTTTTAGTAAACAAAT
A		TGGTGGTACTATTGGTACAG	TCTTTTCATTGGTTTATCCA
GAAAA		GGTGAAGCTGCAAACCCCAG	CCGTTCCAAGAGCCATCAAA
A		CTAACTCCAGTAATTCCTTG	TGGTACGAACATCCAATGAA
AA		AACGCTGAAGTGAAGAGAGA	TTAAGCAAAGACATATTGGT
T		ACTAGGATGAGTAGCAGCAC	TTCCTTATATGTAGCTTTCG
A		CTAACACAATCTACTTCCTA	GTTTCTACTTCTCCCTTTAT
AA	+2	TGTCTTCGAAGAGTAAAAAA	TTGTACTTGGCGGATAATGC
ATA		TGGGCGCTCTTATATCATAT	ATTTATGGGTTCTTTGGCAT
A		TTCCCTTTGCAAATAGTCCT	TTCCAACAATAATAATGTCA

A		TTGGAGAAACCCAGGTGCCT	GGGTCCAGGTATAATATCTA
**T**AAAAA**AA**CA**A**		GCTTAACTGTGCCCTCCATG	TCAAGATATCCACATGTGTTT
G		CACAATAATGAACTTGTTCC	TGTCAAATATTACGGTGAAT
C		AATAGCTTGGCAGCAACAGG	CTAGGATGAGTAGCAGCACG
-	-1	CATACATTGCAGTTTTCGTT	ACTGCTGCATTTGGCGCTTT
C		CCGCCTGCTTCAAACCGCTA	CAATACCTGGACCCACCACA
T		GAGAAACCCAGGTGCCTGGG	TCCAGGTATAATATCTAAGG
ATGTTTC		TCCAAGAGCCATCAAAAAAG	CCGTATCTTAACCTTCTACA
A		TGCTAACATCAAAAGGCCTC	AGGTTCCTTTATTACTTCTT
A		GCTAACAATACCTGGACCCA	CACACCGTGTGCATTCGTAA
A		TCAAATATTACGGTGAATTC	AGTTCTGGGTCGCTTCCATC

type of mutation
substitution
substitution
substitution
substitution
deletion
substitution
substitution
substitution
substitution
substitution
substitution
substitution
deletion
substitution
substitution
substitution
substitution
substitution
substitution
substitution
substitution
substitution
substitution
substitution
substitution
substitution
complex
substitution
complex
substitution
substitution
insertion
complex
substitution

substitution
complex
substitution
substitution
deletion
substitution
substitution
complex
substitution
substitution
substitution

Table S6. Catalog of spontaneous, hydrogen peroxide- and paraquat - induced mutations in subtelomeric ssDNA .
Distance from telomere is relative to the start of non-repetitive subtelomeric DNA.
20 bases on each side flanking a mutation are included.
Sequences are of the top strand of DNA, i.e. the reverse complement of the bottom strand that comprised the 3' s
Separate sheets contain lists of
-mutations induced by hydrogen peroxide and paraquat, detected in *wt, cta1 Δ* strains and *wt , sod1 Δ* strains, re
- spontaneous mutations in *CAN1* ssDNA reporter
- spontaneous and hydrogen-peroxide induced mutations in *rev3 Δ* strains in *CAN1* ssDNA reporter
-spontaneous and hydrogen-peroxide induced mutations in *cta1 Δ rev3 Δ* strains in *CAN1* ssDNA reporter
- spontaneous and paraquat-induced mutations in *sod1 Δ rev3 Δ* strains in *CAN1* ssDNA reporter.
-hydrogen-peroxide induced mutations in *ung1 Δ* strains in *CAN1* and *CAN1-URA3* ssDNA reporter

sDNA overhang formed in temperature-shifted *cdc13-1* cells.

espectively, in *CAN1-URA3* ssDNA reporter;
