# Supplementary material for: Oxidative stress-induced mutagenesis in single-strand DNA occurs primarily at cytosines and is DNA polymerase zeta-dependent only for adenines and guanines
Source: Nucleic Acids Res. 2013 Aug 7;41(19):8995–9005. doi: 10.1093/nar/gkt671 (PMC3799438; doi:10.1093/nar/gkt671)
Supplement: Supplementary Data [file supp_gkt671_nar-01149-f-2013-File011.pdf]

Supplementary Table 1

Combined mutational spectra of spontaneous and oxidative DNA damage in ssDNA reporter

Relevant genotype	Exogenous oxidtive agent	Damaged nucleotide/type of the mutation						Total
		G	C	A	T	indel	Complex	
WT	none	16	5	2	0	5	2	30
WT*	5 mM H_2O_2	3	18	5	2	2	7	37
*cta1**	5 mM H_2O_2	13	22	9	3	7	2	56
*sod1**	150 uM PQ	9	12	4	5	7	4	41
rev3	none	4	5	1	0	13	0	23
rev3	5 mM H_2O_2	0	17	3	1	2	0	23
cta1 rev3	none	8	4	0	1	8	1	22
cta1 rev3	5 mM H_2O_2	2	18	1	0	2	0	23
sod1 rev3	none	5	8	1	1	9	0	24
sod1 rev3	150 uM PQ	6	11	0	1	3	1	22
*ung1**	5 mM H_2O_2	5	20	6	5	4	5	45

*- combined data for *URA3* and *CAN1* loci

Supplementary Table 2
Spectra of spontaneous and oxidative damage-induced substitutions of cytosines and guanines

DNA damaging agent [b]	Relevant genotype	Substiutions [a]											
		cytosine						guanine					
		Endogenous			Damage-induced			Endogenous			Damage-induced		
		C to A	C to T	C to G	C to A	C to T	C to G	G to A	G to T	G to C	G to A	G to T	G to C
peroxide	*WT* *	2	1	2	4	10	4	3	10	3	0	1	2
peroxide	*rev3*	1	4	0	2	12	3	4	0	0	0	0	0
peroxide	*cta1**	n.d.	n.d.	n.d.	5	13	4	n.d.	n.d.	n.d.	0	10	3
peroxide	*cta1 rev3*	0	4	0	2	15	1	2	6	0	0	2	0
paraquat	*sod1* *	n.d.	n.d.	n.d.	2	8	2	n.d.	n.d.	n.d.	1	4	4
paraquat	*sod1 rev3*	1	5	2	1	10	0	1	4	0	0	6	0
peroxide	ung1*	n.d.	n.d.	n.d.	6	10	4	n.d.	n.d.	n.d.	1	3	1

[a] Number of spontaneous and oxidative damage-induced substitutions at positions of cytosine and guanine detected by sequencing of *CAN1* locus of ssDNA repoter in Can^R mutants

[b] 5mM hydrogen peroxide or 150 uM paraquat
(details in Materials and Methods) was used to induce the mutations
represented in the columns " Damage-induced" substitutions

* Data combined for mutations in *URA3* and *CAN1* loci of ssDNA reporter of Can^R Ura$^-$ double mutants

Supplementary Table 3. Role of DNA polymerase eta in oxidative stress-induced mutagenesis in ssDNA [a)]

Relevant genotype	Frequency of Can ^{R} colonies, (x 10^6)	
	No exposure	5mM hydrogen peroxide
WT	72.9	426.5
rad30Δ	98.4	479.5
cta1Δ	65.8	461.5
cta1Δ rad30Δ	75.9	431.0

[a)] Average frequency of Can^R colonies was determined for 8 independent segregants of the same genotype for the strains with ssDNA sub-telomeric reporter as described in Materials and Methods.

Supplementary Table 4. Average Can ^{R} mutation frequencies and standard deviations of average following exposure to 5 mM hydrogen peroxide

	Average frequency of mutation ± standard deviation, x 10 ^{6}			
	Mid-chromosome reporter		Sub-telomeric reporter	
	No treatment	5 mM H_2O_2	No treatment	5 mM H_2O_2
WT	2.0 ±0.90	26.9 ± 17.37	321.6 ± 179.02	634.1 ± 277.91
cta1	4.0 ±1.84	52.3 ± 22.96	319.1 ±206.22	1204.8 ± 405.56
rev3	1.2 ±0.99	4. 3 ± 2.58	29.3 ±9.05	269.2 ± 127.11
rev3 cta1	1.6 ±0.44	6.2 ±0.75	40.6 ±10.71	301.7 ± 98.29

Supplementary Table 5. Heterozygous diploid strains.

Strain	Relevant genotype	Plasmid *	Reference **
hDNP79 [a)]	JFS1325 *sod1:: AUR1*	pAUR112	Takara Bio Inc, Shiga, Japan
hDNP80 [a)]	JFS1326 *sod1:: AUR1*	pAUR112	Takara Bio Inc, Shiga, Japan
hDNP81 [b)]	JFS1327 *sod1:: AUR1*	pAUR112	Takara Bio Inc, Shiga, Japan
hDNP82 [b)]	JFS1328 *sod1:: AUR1*	pAUR112	Takara Bio Inc, Shiga, Japan
hDNP191 [a)]	hDNP79 *rev3::NAT*	pYM17	(1)
hDNP193 [a)]	hDNP80 *rev3::NAT*	pYM17	(1)
hDNP195 [b)]	hDNP81 *rev3::NAT*	pYM17	(1)
hDNP197 [b)]	hDNP82 *rev3::NAT*	pYM17	(1)
hDNP201 [a)]	hDNP191 *cta1::kanMX*	pFA6-KanMX4	(2)
hDNP203 [a)]	hDNP193 *cta1::kanMX*	pFA6-KanMX4	(2)
hDNP205 [b)]	hDNP195 *cta1::kanMX*	pFA6-KanMX4	(2)
hDNP207 [b)]	hDNP197 *cta1::kanMX*	pFA6-KanMX4	(2)

*Plasmid used for PCR amplification of the fragments for the replacement of corresponding gene.

**Reference for the plasmids

[a)] These strains contain mid-chromosome *CAN1-URA3* reporter

[b)] These strains contain sub-telomeric *CAN1-URA3* reporter

1. Janke, C., Magiera, M.M., Rathfelder, N., Taxis, C., Reber, S., Maekawa, H., Moreno-Borchart, A., Doenges, G., Schwob, E., Schiebel, E. *et al.* (2004) A versatile toolbox for PCR-based tagging of yeast genes: new fluorescent proteins, more markers and promoter substitution cassettes. *Yeast*, **21**, 947-962.
2. Wach, A., Brachat, A., Pohlmann, R. and Philippsen, P. (1994) New heterologous modules for classical or PCR-based gene disruptions in Saccharomyces cerevisiae. *Yeast*, **10**, 1793-1808.

Supplementary Table 6. Primers for PCR amplification and sequence of the *CAN1* and *URA3* loci

PCR primers	Sequence	ORF
oDG_77	TCTTGCAAATGCAGCTTCTTC	*URA3*
oDG_78	AAGAACGAAGGAAGGAGCACA	*URA3*
oDG_79	CAAATTCAAAAGAAGACGCCG	*CAN1*
oDG_80	TCCCTTAAACTTTCTTTTCGG	*CAN1*
Sequencing primers		
seqDG_77	TCTTGCAAATGCAGCTTC	*URA3*
seqDG_78	CTCCAGTAGATAGGGAGC	*URA3*
seqDG_80	AAGAACGAAGGAAGGAGC	*URA3*
seqDG_81	CGGGTGTATACAGAATAG	*URA3*
seqDG_83	CAAATTCAAAAGAAGACG	*CAN1*
seqDG_85	TGGGGTCCAGGTATAATA	*CAN1*
seqDG_88	CCCTTAAACTTTCTTTTC	*CAN1*
seqDG_89	AACTCGTCACGAGAGATG	*CAN1*
seqDG_90	AGATTGTGTTAGTTTAGG	*CAN1*
seqDG_91	TTTGACAGGGAACAAGTT	*CAN1*

Supplementary Figure Legends

Figure S1. Exogenous oxidative damage induces mutagenesis in ssDNA. Mutation frequencies in the *CAN1* locus were calculated as described in Materials and Methods. Average mutation frequency measured for at least six independent cultures incubated at 37^0C for 3.5 hours to induce telomere uncapping followed by exposure to 150 μM paraquat (purple bars) or mock treatment (orange bars) are presented. Error bars represent standard deviation of average.

Figure S2. Average percentage of Ura^- colonies among all Can^R colonies per genotype per experimental condition for at least four independently-selected cultures is presented. Error bars represent standard deviation.

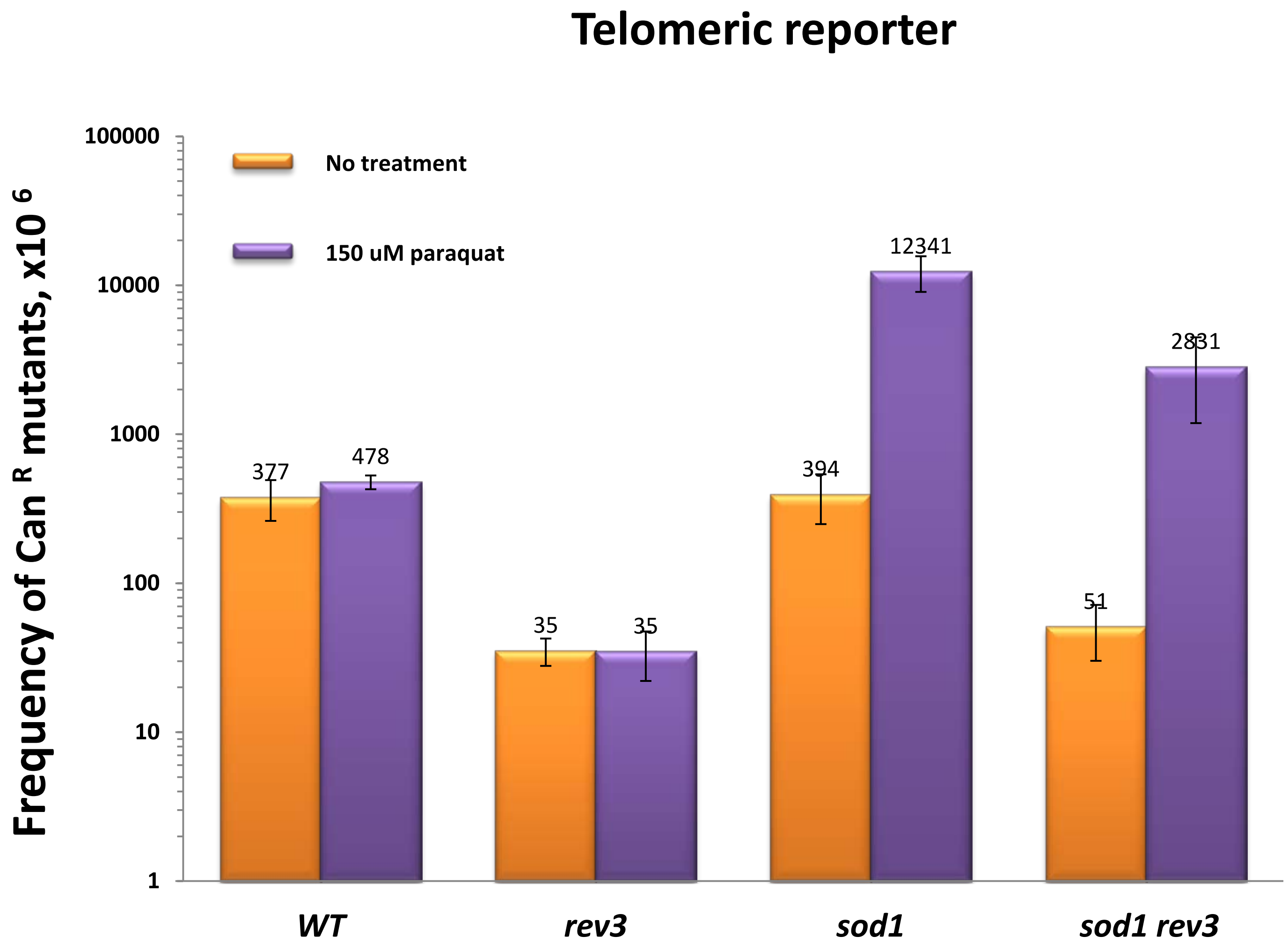

Average survival 100.5% 81.7% 3.7% 5.5%

Figure S1

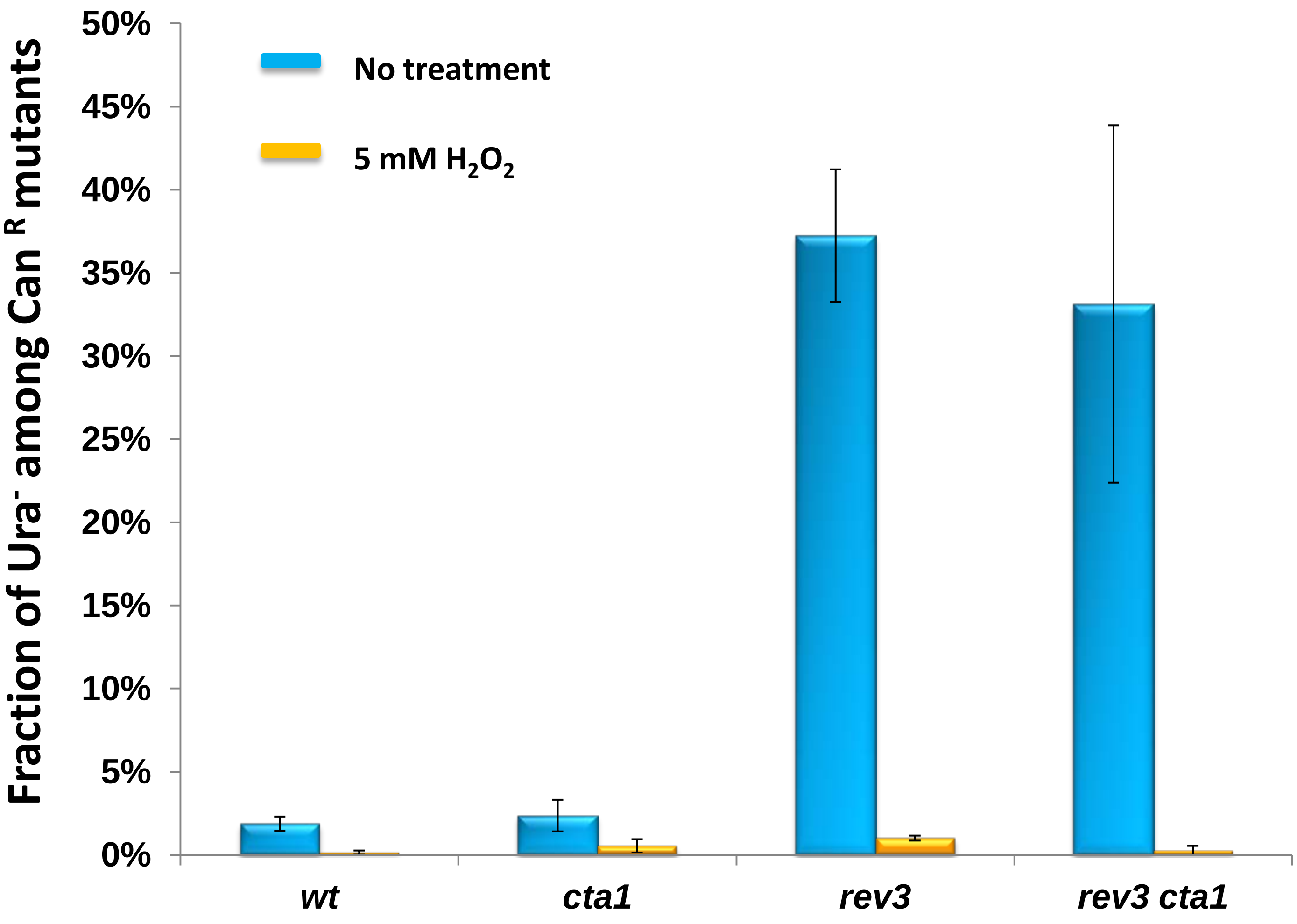

Fraction of Ura- among Can R mutants
50%
45%
40%
35%
30%
25%
20%
15%
10%
5%
0%
No treatment
5 mM H_2O_2
wt
cta1
rev3
rev3 cta1

Figure S2
